# Epithelial to mesenchymal transition influences fibroblast phenotype in colorectal cancer by altering miR‐200 levels in extracellular vesicles

**DOI:** 10.1002/jev2.12226

**Published:** 2022-05-20

**Authors:** Rahul Bhome, Muhammad Emaduddin, Victoria James, Louise M. House, Stephen M. Thirdborough, Massimiliano Mellone, Joeri Tulkens, John N. Primrose, Gareth J. Thomas, Olivier De Wever, Alex H. Mirnezami, A. Emre Sayan

**Affiliations:** ^1^ Cancer Sciences Unit University of Southampton Southampton UK; ^2^ University Surgery University of Southampton Southampton UK; ^3^ School of Veterinary Medicine and Science University of Nottingham Nottingham UK; ^4^ Laboratory of Experimental Cancer Research Department of Human Structure and Repair Ghent University Ghent Belgium

**Keywords:** cancer‐associated fibroblast, colorectal cancer, epithelial to mesenchymal transition, extracellular vesicle, MiR‐200, stroma, Zeb1

## Abstract

Colorectal cancer (CRC) with a mesenchymal gene expression signature has the greatest propensity for distant metastasis and is characterised by the accumulation of cancer‐associated fibroblasts in the stroma. We investigated whether the epithelial to mesenchymal transition status of CRC cells influences fibroblast phenotype, with a focus on the transfer of extracellular vesicles (EVs), as a controlled means of cell–cell communication. Epithelial CRC EVs suppressed TGF‐*β*‐driven myofibroblast differentiation, whereas mesenchymal CRC EVs did not. This was driven by miR‐200 (miR‐200a/b/c, ‐141), which was enriched in epithelial CRC EVs and transferred to recipient fibroblasts. Ectopic miR‐200 expression or *ZEB1* knockdown, in fibroblasts, similarly suppressed myofibroblast differentiation. Supporting these findings, there was a strong negative correlation between miR‐200 and myofibroblastic markers in a cohort of CRC patients in the TCGA dataset. This was replicated in mice, by co‐injecting epithelial or mesenchymal CRC cells with fibroblasts and analysing stromal markers of myofibroblastic phenotype. Fibroblasts from epithelial tumours contained more miR‐200 and expressed less *ACTA2* and *FN1* than those from mesenchymal tumours. As such, these data provide a new mechanism for the development of fibroblast heterogeneity in CRC, through EV‐mediated transfer of miRNAs, and provide an explanation as to why CRC tumours with greater metastatic potential are CAF rich.

## 1 INTRODUCTION

Colorectal cancer (CRC) is the third most common malignancy in the world, with global incidence predicted to surpass two million, and mortality one million, by 2030 (Arnold et al., 2017). Over 90% of deaths in those with solid tumours such as CRC are due to metastasis (Mehlen & Puisieux, 2006). This highlights the importance of studying the biology of CRC progression, in order to identify better prognostic markers and devise targeted treatments. Recent efforts have led to the molecular subtyping of CRC. A globally recognised classification system defined the consensus molecular subtypes (CMS) of CRC and stratified patients into four subgroups (CMS1‐4) (Guinney et al., 2015). Among these, CMS4 has the worst prognosis and is associated with metastasis and chemoresistance. Importantly, this subtype did not differ from others in terms of mutational burden, somatic copy number alterations or CpG island methylation, but rather the presence of stromal cells and a mesenchymal (or transforming growth factor (TGF)‐β activation) gene expression signature. Therefore, there seems to be an important link between metastatic cells and stromal phenotype in CRC.

The tumour microenvironment is considered to be an ecosystem of cancer and stromal elements (Bhome et al., 2015). Fibroblasts are the predominant cell type in the stroma and are responsible for the production of growth factors, cytokines, chemokines, enzymes and extracellular matrix (Kalluri, 2016; Kalluri & Zeisberg, 2006). Importantly, the phenotype of myofibroblastic cancer‐associated fibroblasts (CAFs) is associated with oncological outcome in CRC (Herrera et al., 2013). The presence of myofibroblasts is a marker of poor prognosis, not only in CRC but also in several other solid tumours, such as prostate, head and neck and pancreas (Ayala et al., 2003; Marsh et al., 2011; Sinn et al., 2014; Tsujino et al., 2007).

In the classical model of myofibroblast activation, TGF‐β stimulates the expression of α‐smooth muscle actin (α‐SMA) in fibroblasts, conferring increased contractility and facilitating wound healing (Desmoulière et al., 1993). Cancer is considered to be a ‘wound that does not heal’, in which myofibroblasts are thought to remain persistently activated (Dvorak, 1986), resulting in upregulation of growth factors and pro‐inflammatory cytokines (Orimo et al., 2001). This has been shown to influence stromal remodelling (Lu et al., 2011; Otranto et al., 2012), facilitate collective and individual cancer cell migration (Gaggioli et al., 2007; Gascard & Tlsty, 2016) and promote chemoresistance (Ireland et al., 2016; Müerköster et al., 2008). The CRC microenvironment is TGF‐β‐rich, with elevated circulating levels detectable in CRC patients (Markowitz et al., 1995; Tsushima et al., 2001). Although *TGFB1* expression is comparably lower in Stage I disease, there is no significant difference between Stages II/III and Stage IV, suggesting that it may be involved in initial disease progression but not directly linked to metastasis (Calon et al., 2012). Considering that a significant proportion of colorectal tumours have inactivating mutations in the TGF‐β pathway, the effect of TGF‐β is unlikely to be directed to cancer cells and is more likely to influence the stroma (Cancer Genome Atlas Network, 2012).

It is important to note that studies of stromal heterogeneity have identified several different CAF populations, only some of which are α‐SMA‐positive, with each having a different contribution to cancer progression (Costa et al., 2018; Li et al., 2017; Sugimoto et al., 2006). This raises the fundamental question of how α‐SMA‐positive CAF populations are formed in some tumours but not in others. This is especially intriguing in CRC, where there is an excess of local TGF‐β in the majority of tumours (Calon et al., 2012). The histological organisation of CAFs and cancer cells provides certain clues, with the co‐localisation of myofibroblasts and invasive carcinoma cells reported in various solid tumours (Kawashiri et al., 2009; Öhlund et al., 2017; Takatsuna et al., 2016). In CRC, the most lethal CMS4 subtype produces a stroma rich in α‐SMA‐positive myofibroblasts (Becht et al., 2016; Guinney et al., 2015). These observations suggest that the phenotype of cancer cells in the primary tumour shapes the stromal landscape, and in particular, that metastatic capability promotes myofibroblast accumulation. However, the exact mechanism for this is currently unknown.

A key molecular feature which distinguishes metastatic and non‐metastatic carcinoma cells is epithelial to mesenchymal transition (EMT). This programme of cellular differentiation is characterised by loss of epithelial identity and acquisition of mesenchymal features such as increased motility. EMT is regulated by the ZEB, Snail and Twist families of transcription factors (TFs) (Hay & Zuk, 1995; Kalluri & Neilson, 2003; Kalluri & Weinberg, 2009). Of note, the CMS4 subtype of CRC represents an EMT‐associated (mesenchymal) molecular signature (Guinney et al., 2015). Therefore, the relationship between EMT and myofibroblast accumulation warrants an investigation for causality; specifically, the signals that tumour cells provide to alter stromal phenotype.

Tumour and stromal cells communicate by several mechanisms, such as cell–cell contact and secretion of soluble factors (Labernadie et al., 2017; Orimo et al., 2005). The discovery that extracellular vesicles (EVs) transmit functional nucleic acids and other macromolecules between cells, has placed a spotlight on this method of communication in recent years (Valadi et al., 2007). Of note, microRNAs (miRNAs) are a stable component of EV cargo, and evidence suggests that loading miRNAs into EVs is a selective process (Bhome et al., 2018; Guduric‐Fuchs et al., 2012; Koppers‐Lalic et al., 2014; Kosaka et al., 2010; Villarroya‐Beltri et al., 2013).

The characterisation of stromal EV cargo and its effect on CRC cells has previously been described (Bhome et al., 2017). In the present study, we sought to investigate the reverse relationship. Specifically, we asked whether the EMT status of CRC cells is a determinant of myofibroblast phenotype, and whether exchange of EV miRNAs mediates the interplay between these cell types in the presence of TGF‐β.

## 2 METHODS

A description of cloning, cell lines (including the generation of stably transduced cell lines), MLEC assay, western blotting, transmission electron microscopy (TEM), nanoparticle tracking analysis (NTA), RNA extraction and RT‐qPCR array/ assays is provided in Supplementary Methods.

### 2.1 EV isolation

EVs were isolated by differential ultracentrifugation, using an optimised version of our previous method (Bhome et al., 2017). Briefly, CRC cells were grown to 70% confluence in three 175 cm^2^ flasks (4‐8 × 10^7^ cells), at which point the growth medium was replaced with equivalent medium supplemented with EV‐depleted FBS (supernatant of FBS, ultracentrifuged at 100,000 × *g* for 16 h). After 72 h, conditioned medium was harvested and centrifuged at 400 × *g* for 5 min to pellet floating cells, followed by 2000 × *g* for 10 min to pellet cellular debris. The supernatant was then centrifuged at 10,000 × *g* for 30 min to pellet apoptotic bodies and microparticles (>1μm). The resulting supernatant was then filtered (0.22 μm) and ultracentrifuged at 100,000 × *g* for 75 min at 4°C using a TFT 50.38 rotor (ThermoFisher). The resulting EV pellets were pooled, washed with PBS and ultracentrifuged again at 100,000 × *g*. The final EV pellet was solubilised in 200 μl PBS and stored at ‐80°C.

Additionally, EVs were isolated by an alternative validated technique, combining precipitation and size exclusion chromatography (SEC), using the Exo‐Spin kit (cat no. EXO1; Cell Guidance Systems), as per manufacturer instructions (Kavanagh et al., 2017). Conditioned medium was centrifuged at 300 × *g* for 10 min and then 16,000 × *g* for 30 min to exclude cells and cellular debris. Exo‐Spin buffer was added to the supernatant in a 2:1 ratio and incubated overnight at 4°C. The mixture was then centrifuged at 16,000 × *g* for 1 h and the resulting EV pellet was resuspended in 1 ml PBS. Exo‐Spin columns were equilibrated at room temperature using PBS. The EV suspension was then applied to the column and the initial flow‐through was discarded. PBS was then applied and 1 ml fractions were collected. As recommended by the manufacturer, fractions 7–12 were collected, pooled and then qualitatively and quantitatively analysed.

We have submitted all relevant data from our experiments to the EV‐TRACK knowledgebase (EV‐TRACK ID: EV190029) (Van Deun et al., 2017).

### 2.2 Cellular EV uptake

EVs were isolated from CRC cells and labelled with Vybrant DiO (cat no. V22886; ThermoFisher). We identified that labelling EVs after isolation significantly decreased the probability of DiO binding to protein or lipid aggregates (Van Der Vlist et al., 2012). Therefore, EV labelling was not performed on live cells but as part of the final wash steps during EV isolation. Furthermore, a much lower concentration of DiO (1 in 10,000) was used to reduce non‐specific binding. After labelling, DiO‐EVs were washed again with PBS and ultracentrifuged to remove excess dye. As a control (for dye aggregates), we labelled EV‐depleted medium with the same concentration of DiO and processed it like an EV preparation (i.e., ultracentrifugation).

MRC5 fibroblasts (4 × 10^5^) were conditioned with labelled EVs at a concentration of 0.5 × 10^9^ particles/ml, or an equivalent volume of DiO‐labelled medium (control), for 24 h, in 6‐well plates. At 24 h, fibroblasts were washed twice with PBS and the DiO signal was detected using an inverted bright‐field fluorescence microscope.

### 2.3 Detection of labelled EVs by flow cytometry

MRC5 fibroblasts were conditioned with DiO‐ or DiD‐labelled EVs (0.5 × 10^9^ particles/ml) or DiO/ DiD‐labelled medium (see above) for 24 h (Vybrant DiD; cat. no. V22887; ThermoFisher). Fibroblasts were then dissociated into single cells (Trypsin‐EDTA 0.25%; cat. no. T4049; Sigma) and live cells analysed by flow cytometry (FACS Calibur). Following duplet exclusion, the presence of intracellular DiO/ DiD‐positive particles was assessed in the FL1/ FL4 channels, respectively.

### 2.4 Conditioning of fibroblasts with CRC EVs and subsequent myofibroblast differentiation

To mimic the constant exposure of fibroblasts to EVs *in vivo*, fibroblasts were conditioned with CRC EVs every day for 5 days, replenishing the media every day. 4 × 10^5^ MRC5 fibroblasts or normal colon fibroblasts (NCFs) were seeded into 6‐well plates, in EV‐free medium. The next day (day 0), the medium was changed and EVs were added at a concentration of 1.5 × 10^9^ particles/ml. The medium was changed, and fresh EVs were added in a similar fashion on days 2–5. Cells were passaged on day 5, with one third collected for RNA analysis where necessary. On day 6, the medium was changed to a low serum (0.1% FBS) EV‐free medium, and on day 7, TGF‐β was added at a concentration of 2 ng/ml (Thannickal et al., 2003; Yang et al., 2012). Low serum conditions were used because serum has been shown to influence fibroblast contractility and differentiation in multiple models (Galie et al., 2011; Jester et al., 1996). An equal number of wells were untreated, as controls. Cells were collected on day 9 for western blotting.

Transient transfection of fibroblasts with miR‐200 mimics, *ZEB1* siRNA or the *ZEB1 3′UTR* luciferase construct is detailed in Supplementary Methods.

### 2.5 Analysis of myofibroblast differentiation in EV‐high and EV‐low fibroblasts

MRC5 fibroblasts were conditioned with fluorescently labelled (DiO) epithelial (DLD1) and mesenchymal (SW480) EVs and stimulated with TGF‐β as described above. Cells were then sorted using FACS Aria (BD Biosciences). As more than 95% of EV‐conditioned cells registered positive, we sorted EV‐high (70–100th centile) and EV‐low (0–30th centile) populations, rather than purely positive and negative populations. Cells were collected in DMEM, pelleted and stored at ‐80°C prior to RNA extraction and quantification of miR‐200, *ACTA2* and *FN1* by qPCR.

### 2.6 RNA labelling and subsequent isolation from fibroblasts

RNA was labelled in MRC5, DLD‐1 and SW480 cells, from which EVs were isolated and used to condition MRC5 cells. 5EU labelling of nascent RNAs was achieved by the addition of 5EU to the cell culture media at a final concentration of 0.4 mM for 24 h. To isolate 5EU‐labelled miRNAs from recipient cells, the Click‐iT Nascent RNA Capture Kit (cat no. C10365; ThermoFisher) was used, following the manufacturer's recommended protocol. Unlabelled controls were used to determine the background level of RNA recovery of the precipitation step. 5EU‐labelled RNA was used directly for RT‐qPCR.

### 2.7 MiRNA and gene expression in human CRC dataset

Three hundred and four tumour samples with matched miRNA and gene logCPM values were identified in the TCGA dataset (https://portal.gdc.cancer.gov/). A panel of CRC tumour cell‐associated genes was selected, to include common colonic epithelial markers (*CDH1, CDH3, KRT18, KRT19, KRT20*, *CEACAM1, CEACAM5, MUC1, MUC2, MUC6*) (Byrd & Bresalier, 2004; Majumdar et al., 2012). Similarly, a panel of myofibroblast‐associated genes (*DES, ACTA2, TNS1, PDGFRA, S100A4, VCL, VIM, PALLD, FN1, POSTN, FAP, PDGFRB*) was selected based on a consensus of previous studies (Lebleu & Kalluri, 2018; Shiga et al., 2015). The panel of miRNAs was selected based on those which had the highest epithelial EV to mesenchymal EV ratio from the miRNA array (described in Supplementary Methods). The top 20 were selected and expanded to include complete families (e.g., let‐7a‐1, a‐2, a‐3, let‐7b and miR‐200a/b/c, ‐141). Correlations between miRNA and gene expression were quantified by Pearson correlation coefficient, r, and statistical significance determined by Student asymptotic *p* values.

### 2.8 *In vivo* study

All mice were housed in a specific pathogen‐free facility at the University of Southampton and given a commercial basic diet and water *ad libitum*. Six 6–8 week‐old CD‐1 nude mice were injected subcutaneously (into both flanks) with 7.5 × 10^5^ SW480‐control cells and 2 × 10^6^ PKH‐labelled MRC5 fibroblasts (mesenchymal tumours; *n* = 12), or, 7.5 × 10^5^ SW480‐ZEB1 knockdown (ZKD) cells and 2 × 10^6^ MRC5 PKH‐labelled fibroblasts (epithelial tumours; *n* = 12). Prior to injection, cells were mixed, pelleted and resuspended in 100 μl serum‐free medium, to which an equal volume of Matrigel (cat no. 356237; Corning) was added. At 2 weeks, animals were sacrificed and the tumours were excised. Immunohistochemical staining, single cell dissociation, flow sorting and RT‐qPCR pertaining to the *in vivo* study are described in Supplementary Methods.

### 2.9 Statistical analysis

Where individual images (microscopy, western blotting and flow cytometry) are displayed, these are representative of at least three separate experiments. Graphics represent the mean ± SEM, unless otherwise stated. *TGFB1* expression for CRC cell lines in the CCLE cohort was compared by Mann–Whitney *U* Test. RT‐qPCR was performed in triplicate and differences in mean relative values were tested by a two‐tailed, unpaired *t*‐test. Where applicable, proportions of positively and negatively gated cells from flow cytometry were compared by Fisher's exact test (two‐tailed). Luciferase assays were performed in triplicate and differences in mean relative values were tested by two‐tailed unpaired *t*‐test. The threshold level of significance was set at 0.05 for all statistical tests. The level of statistical significance was denoted by *p *< 0.05 (*); *p *< 0.01 (**); and *p *< 0.001 (***).

## 3 RESULTS

### 3.1 *In vitro* CRC models represent a spectrum of EMT phenotypes

**FIGURE 1:**
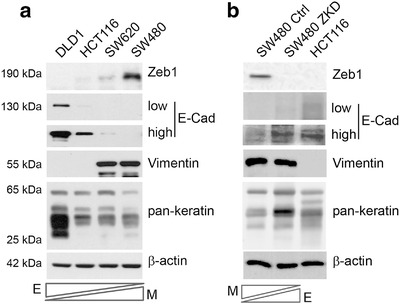
EMT status of CRC models. (a) Assessment of EMT marker expression in a spectrum of epithelial and mesenchymal CRC cell lines by western blotting. (b) EMT marker expression in the SW480 MET model (SW480‐ZKD cells), where HCT116 cells are shown as a positive control. Low and high exposures for E‐cadherin in both panels. *E‐*epithelial and *M‐*mesenchymal. Representative of three separate experiments.

First, we sought to identify CRC cell lines which represent tumour heterogeneity in terms of epithelial or mesenchymal characteristics. Gene expression data for 52 CRC cell lines from the CCLE cohort of the Gene Expression Atlas (https://www.ebi.ac.uk/gxa/home) suggest that the majority of CRC cell lines are epithelial (expressing variable but detectable E‐cadherin (*CDH1*) and keratins (*KRT18, KRT20*)), with only a small minority (5/52) purely expressing mesenchymal markers (Vimentin (*VIM*) and *ZEB1*; (Figure S1A) (Papatheodorou et al., 2018). To reflect a spectrum of EMT phenotypes, four CRC cell lines were selected from our repository. DLD1 cells showed the most epithelial phenotype (high keratin and E‐cadherin, no vimentin or ZEB1), HCT116 and SW620 cells expressed reduced E‐cadherin, and were considered metastable (showing both epithelial and mesenchymal features), whereas SW480 cells were considered mesenchymal as they had high ZEB1 and vimentin, no‐E‐cadherin and very low levels of keratins (Figure 1a). In addition, we knocked down *ZEB1* in SW480 cells (SW480‐ZKD) to generate an isogeneic but more epithelial cell line, representing a mesenchymal to epithelial transition (MET) model (Figure 1b), as described previously (Burk et al., 2008). To further validate this model, exogenous murine *zeb1* cDNA (with sequence disparity at the human *ZEB1* shRNA binding site) was transfected into SW480‐ZKD (human) cells. These cells underwent EMT, showing specificity of the shRNA, and the knockdown phenotype could be rescued (Figure S1B). Accordingly, we used these cell lines, with defined EMT status, to mimic the heterogeneity of CRC tumours.

CRC is a TGF‐β rich cancer, with CRC tumours and cell lines expressing more TGF‐β than normal colonic epithelium (Hawinkels et al., 2014). There is large variation in *TGFB1* expression CRC cell lines in the CCLE cohort; however, the four cell lines selected for this study fall in a narrow range, despite clear differences in EMT status (Figure S1C). In keeping with this, our isogenic MET model (SW480 and SW480‐ZKD cells) also showed no significant difference in *TGFB1* expression (data not shown) or activity, as assessed by MLEC assay (Figure S1D). Therefore, we were in a position to investigate the effect of EMT on fibroblast phenotype via EVs, in a context where TGF‐β was present but not significantly different between the CRC models that were used.

### 3.2 CRC cells produce EVs which are transferred to fibroblasts

**FIGURE 2:**
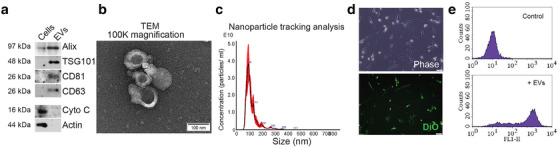
Characterisation and transfer of CRC EVs. (a) Endosomal (Alix, TSG101) and tetraspanin (CD63, CD81) marker expression in HCT116 cells and EVs. The mitochondrial protein, cytochrome C, was used as a negative EV marker. Representative of three separate experiments. (b) Transmission electron micrograph of HCT116 EVs at 100,000× magnification. Scale bar represents 100 nm. Representative of three EV preparations. (c) Nanoparticle tracking analysis of HCT116 EVs from five separate videos, each 90s duration. (d) Visualisation of DiO‐labelled HCT116 EVs within MRC5 fibroblasts after 24 h conditioning. Phase contrast and fluorescence images are shown. Scale bar represents 50 μm. Representative of three experiments. (e) Detection of DiO‐labelled HCT116 EVs in MRC5 fibroblasts by flow cytometry. Representative histograms for control (DiO‐labelled medium) and EV‐conditioned fibroblasts from three experiments.

Next, we wanted to determine the paracrine effect of epithelial and mesenchymal CRC cells on fibroblasts, with a focus on EVs, as ubiquitously produced and selectively loaded components of the secretome (Bhome et al., 2018; Janas et al., 2015). CRC EVs were isolated from cell culture conditioned media by differential ultracentrifugation. Isolated vesicles displayed enrichment in endosomal markers (Alix, TSG101) and tetraspanins (CD63, CD81) but were lacking in markers for other organelles such as mitochondria (Cytochrome C; Figure 2a) and were homogenous in morphology (Figure 2b), with a modal size in the range of 90–130 nm (Figure 2c). Epithelial and mesenchymal CRC cells produced EVs with comparable size (Figure S2A) and a similar number of EVs per cell (Figure S2B). To assess the general effect of CRC EVs on fibroblasts, we chose one of the most commonly used fibroblast lines, namely MRC5 (obtained from human embryonic lung), which can undergo over 40 passages before senescence (Jacobs et al., 1970). These cells have a defined TGF‐β response and have been shown to behave similarly to primary colonic fibroblasts (Bullock et al., 2013). Initially, we wanted to assess if EVs from different CRC cells were taken up by fibroblasts with similar efficacy; therefore, we fluorescently labelled EVs and conditioned fibroblasts for 24 h. Following this, fibroblasts were washed to remove non‐internalised EVs and fluorescence signal was detected by microscopy (Figure 2d) and flow cytometry (Figure 2e). Epithelial and mesenchymal EVs were shown to be taken up with similar efficiency by fibroblasts (Figure S2C). Therefore, epithelial and mesenchymal CRC cells produce EVs of similar characteristics, which are taken up by fibroblasts in similar quantities.

### 3.3 Epithelial and mesenchymal CRC EVs produce different responses in fibroblast signalling pathways

Having established that CRC cells transfer EVs to fibroblasts, we then asked whether EVs from epithelial and mesenchymal CRC cells could differently influence fibroblast biology. We initially investigated ERK and AKT pathways, which have wide ranging implications for cell fate and have been shown to be important in CAF‐related tumour progression (Nho et al., 2005; Rosenfeldt & Grinnell, 2000; Serrano et al., 1997; Trimboli et al., 2009). Before conditioning fibroblasts with EVs, we sought to document ERK and AKT activity in donor CRC cells. All CRC cell lines used in this study were *KRAS* mutant; therefore, they registered ERK pathway activity (p‐ERK positive), albeit at different levels (Figure S3A, B). Next, we applied EVs from CRC cells in our panel to fibroblasts, at different concentrations, in order to observe any dose‐dependent trends in ERK and AKT activity.

Conditioning of fibroblasts with epithelial but not mesenchymal EVs, attenuated ERK activity, even at the lowest tested concentration (Figure S3C), implying that epithelial CRC cells have a specific inhibitory effect on fibroblast ERK pathway. AKT activity in fibroblasts increased in a dose‐dependent manner with HCT116 and SW620 EVs but not DLD1 or SW480, therefore, appearing unrelated to EMT status of donor CRC cells (Figure S3C). A similar pattern of results on ERK activity was seen using epithelial and mesenchymal EVs from the SW480‐ZKD model, justifying our initial observations (Figure S3D). Of note, cellular levels of ERK and AKT proteins and their phosphorylated isoforms in donor CRC cells, did not correlate with the changes observed in EV‐recipient fibroblasts. For example, DLD1 and SW480 cells expressed a similar amount of phospho‐ERK, but ERK activity in fibroblasts conditioned with DLD1 and SW480 EVs was different. This suggests that the demonstrated effects of EVs on fibroblasts are unlikely to be the result of EV‐mediated transfer of proteins (i.e., phospho‐ERK or mutant KRAS protein) or mRNA (i.e., mutant *KRAS* mRNA).

### 3.4 MiR‐200 distinguishes CRC cells and EVs by EMT status

**FIGURE 3:**
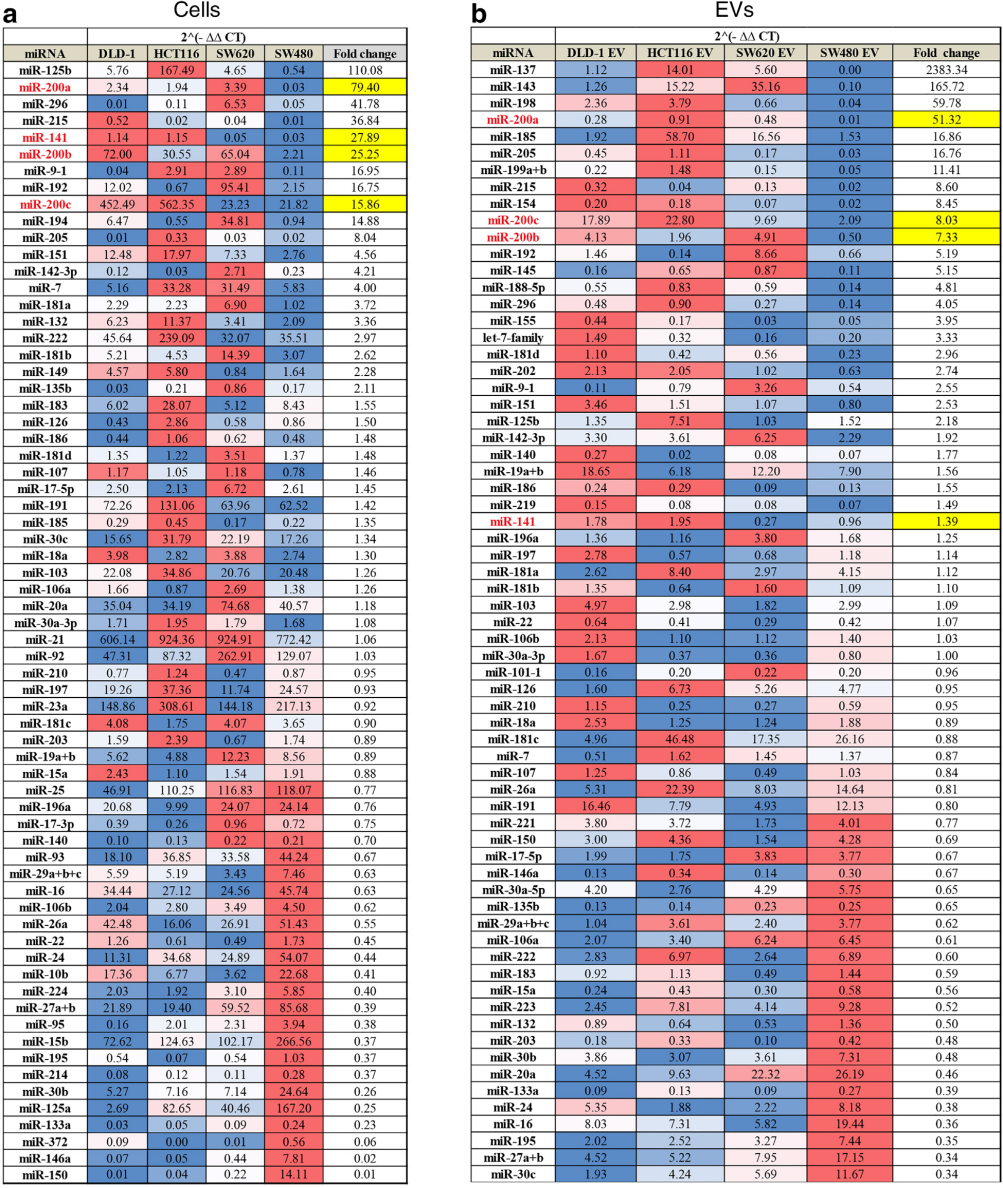
Relative abundance of miRNAs in epithelial and mesenchymal CRC cells and EVs by qPCR array. (a) CRC cells. (b) CRC EVs. CT values were normalised to the geometric mean of all values for that sample. Combined mean 2^(‐ΔΔCT)^ values from three biological replicates for epithelial cells or EVs (DLD‐1, HCT116 and SW620) were compared with mesenchymal (SW480) cells or EVs for each miRNA, to generate fold changes. MiRNAs for which 2^(‐ΔΔCT)^ values were less than 0.1 in all samples were excluded. Fold changes for each miRNA are represented on a blue‐white‐red (low‐median‐high) colour scale. Fold changes for miR‐200 family members are highlighted in yellow.

**FIGURE 4:**
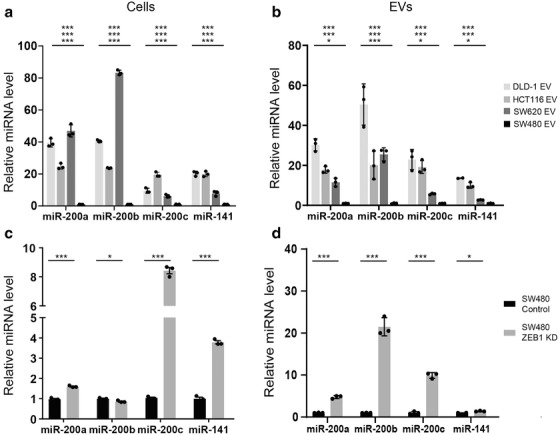
MiR‐200 levels in epithelial and mesenchymal CRC cells and EVs. (a) CRC cells. (b) CRC EVs. (c) SW480‐ZKD cells (SW480‐MET model). (d) SW480‐ZKD EVs. MiRNA levels were normalised to miR‐423‐5p, calculated from the triplicate of CT values, using the ΔΔCT method, and expressed relative to SW480 or SW480 control cells or EVs, which were assigned the value 1. Statistical significance was determined by two‐tailed unpaired *t*‐test (*p < 0.05; **p < 0.01; ***p < 0.001). In (a) and (b), statistical significance is shown for DLD‐1, HCT116 and SW620, compared to SW480 (from top to bottom, respectively). Values plotted are the means of three technical replicates from three experiments.

Since the data indicated that protein or mRNA transfer was less likely to be responsible for the observed effects, we focussed on miRNA content of EVs. MiRNAs are the most stable EV cargo and evidence suggests they may be selectively loaded into EVs (Bhome et al., 2018; Janas et al., 2015). Therefore, we investigated miRNA levels in CRC cells and in their respective EVs using a miRNA array. MiRNAs were ranked according to ratio of abundance in epithelial compared to mesenchymal cells or EVs (Figure 3a, b). Our intention was to understand the differences observed in Figure S3, as well as finding a reason why the CMS4 (mesenchymal) subtype of CRC is CAF rich. Cellular and EV miRNA profiles from the same cell are known to differ (Bhome et al., 2018) due to selective loading; however, miR‐200 family members (miR‐200a/b/c, ‐141) were commonly and consistently more abundant in epithelial cells and EVs compared to their mesenchymal counterparts (Figure 3a, b). Validation experiments confirmed findings from the array in the four CRC cell lines (Figure 4a, b) and also the MET (SW480‐ZKD) model (Figure 4c, d).

### 3.5 CRC EVs deliver miR‐200 to fibroblasts

Having identified miR‐200, perhaps not surprisingly, as a consistent differentiator between epithelial and mesenchymal cells and EVs, we investigated whether this family of miRNAs could be transferred from CRC cells to fibroblasts. To that end, we transfected miR‐200 inhibitors (antagomiRs) into a donor epithelial CRC cell line (DLD‐1) and investigated the transfer of EVs obtained from these cells to fibroblasts. Transfection of antagomiRs led to a significant reduction in cellular (donor) miR‐200a/b/c and ‐141 (Figure S4A). Importantly, EVs obtained from these cells had reduced abundance of all these miRNAs compared to control (Figure S4B). When incubated with these EVs, fibroblasts registered reduced levels of all miR‐200 family members (Figure S4C), suggesting that EV transfer determines miR‐200 levels in recipient fibroblasts.

**FIGURE 5:**
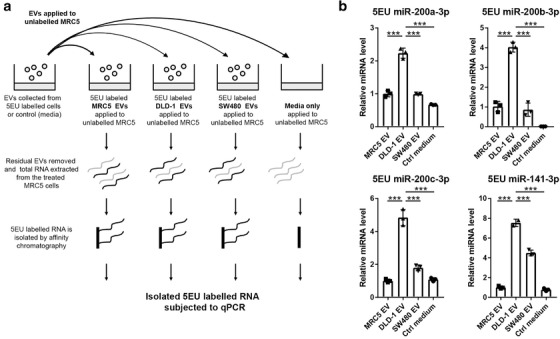
(a) Schematic of RNA pull down experimental set‐up. Nascent RNA was labelled in donor cells or cell‐free control media, from which EVs were isolated and transferred to MRC5 fibroblasts. Labelled RNA was captured from recipient fibroblasts and probed for miR‐200. (b) MiR‐200 levels in 5EU labelled RNA from recipient fibroblasts. MiRNA levels were normalised to miR‐423‐5p, calculated from the triplicate of CT values, using the ΔΔCT method, and expressed relative to MRC5 cells conditioned with MRC5 (self) EVs, which were assigned the value 1. Values plotted are means of three technical replicates from three experiments. Statistical significance was determined by two‐tailed unpaired *t*‐test (***p < 0.001).

Next, we wanted to determine if the changes in miRNA levels in recipient fibroblasts were a result of miRNA transfer through EVs or through upregulation of endogenous miRNAs in fibroblast. To achieve this, we labelled nascent RNAs in donor cells using 5EU, isolated their EVs and applied them to fibroblasts. Labelled RNAs were pulled down in fibroblasts using affinity chromatography and probed for miR‐200, as described in Figure 5a. Epithelial CRC (DLD‐1) EVs were able to deliver more exogenous miR‐200a/b/c and ‐141 to fibroblasts, compared to mesenchymal CRC (SW480) EVs or fibroblast (MRC5) EVs (Figure 5b). These results suggest that the increased miR‐200 in fibroblasts conditioned with epithelial CRC EVs is coming from an exogenous source, rather than upregulation of endogenous miR‐200 as a bystander effect of EV treatment.

### 3.6 EV‐regulated miR‐200/ZEB1 axis controls myofibroblast differentiation

**FIGURE 6:**
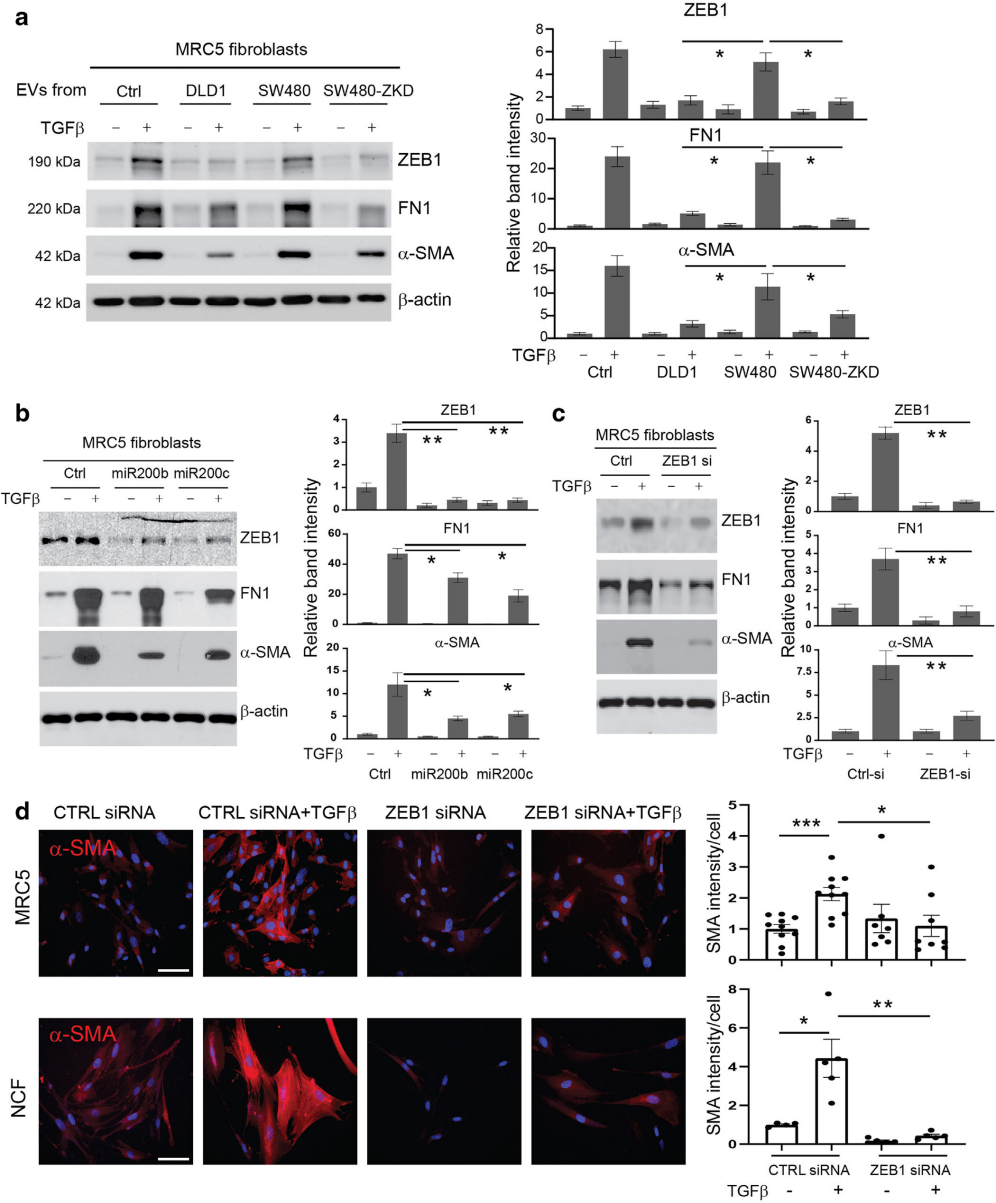
Effects of EVs and miR‐200 on myofibroblast differentiation. Protein expression of ZEB1, α‐SMA and fibronectin by western blotting in MRC5 fibroblasts (with or without TGF‐β stimulation): (a) Conditioning with CRC EVs (epithelial or mesenchymal) at a concentration of 1.5 × 10^9^ EVs/ml (quantified using Nanosight); (b) transfection with miR‐200b/c; (c) transfection with *ZEB1* siRNA. For Panels (a–c), band intensities are relative to β‐actin and normalised to the first lane of the blot which was given the value 1. Values plotted are from three independent experiments. (d) Immunocytochemistry of MRC5 and NCFs (control and ZEB1 siRNA‐transfected) before and after TGF‐β stimulation. Scale bars represent 100 μm. Staining intensity per cell (relative to control‐transfected/ TGF‐β unstimulated fibroblasts) is shown on the right. The graph represents individual data points from three independent experiments. Statistical significance (a‐d) was determined by two‐tailed unpaired *t*‐test (*p < 0.05; **p < 0.01; ***p < 0.001).

Then, we asked whether EV‐mediated changes in miR‐200 levels in recipient fibroblasts had any effect on myofibroblastic phenotype. To approximate physiological conditions, we used primary normal colonic fibroblasts (NCFs) (Bhome et al., 2017) in addition to MRC5. EVs from DLD1 (epithelial) or SW480 (mesenchymal) cells were used to condition fibroblasts every day for 5 days, to simulate the *in vivo* exposure of fibroblasts to constant but small quantities of EVs. Epithelial but not mesenchymal EVs, increased total miR‐200 levels in recipient fibroblasts (Figure S4D). To investigate whether EV‐mediated changes in miR‐200 levels influence myofibroblast phenotype, we utilised a TGF‐β‐driven myofibroblast differentiation assay (Desmoulière et al., 1993). Control fibroblasts (no EVs) stimulated with TGF‐β, showed a significant increase α‐SMA and fibronectin expression (Figure 6a). In these conditions, ZEB1 expression was upregulated in fibroblasts as part of the TGF‐β response (Postigo, 2003; Postigo, 2003). A similar pattern was observed when fibroblasts were conditioned with mesenchymal EVs (miR‐200 low) (Figure 6a). Conversely, in the presence of epithelial EVs (miR‐200 high), the TGF‐β‐mediated increase in myofibroblast markers such as α‐SMA and fibronectin was attenuated (Figure 6a). In these conditions, ZEB1 induction was also abrogated. We performed the same experiment using normal colonic fibroblasts (NCFs), with similar results (Figure S4E).

There are reports suggesting that ultracentrifugation may precipitate free miRNAs, stabilised in protein complexes such as Argonaute‐2 (Van Deun et al., 2014). To exclude the possibility that extra‐vesicular miRNA complexes were confounding our results, we used size exclusion chromatography (SEC) to isolate EVs and exclude molecular contaminants. SEC produced EVs with modal size 100–160 nm, with epithelial and mesenchymal CRC cells producing similar sized EVs (Figure S4F). Similar to EV isolation by ultracentrifugation, epithelial EVs (DLD1 and SW480‐ZKD) contained more miR‐200b and ‐200c than mesenchymal EVs (SW480) (Figure S4G). Conditioning with epithelial but not mesenchymal EVs attenuated TGF‐β‐driven myofibroblast differentiation (Figure S4H). Therefore, regardless of isolation technique, CRC EVs from epithelial cells had a consistent inhibitory effect on myofibroblast differentiation.

To confirm that the effects seen were EV‐dependent, fibroblasts were conditioned with fluorescently labelled (DiO) CRC EVs (epithelial or mesenchymal), stimulated with TGF‐β and sorted into EV^high^ (70–100th centile fluorescence intensity) and EV^low^ (0–30th centile) populations, to distinguish fibroblasts with high and low concentrations of internalised EVs (Figure S5A). Fibroblasts containing a high concentration of epithelial EVs had greater amounts of miR‐200b and ‐200c than fibroblasts containing a low concentration of epithelial EVs (Figure S5B). However, there was no difference in miR‐200 levels in fibroblasts with high or low concentrations of mesenchymal EVs. Baseline and TGF‐β‐stimulated *ACTA2* (α‐SMA) and *FN1* (fibronectin) levels were reduced in fibroblasts with a high epithelial EV concentration compared to those with a low epithelial EV concentration (Figure S5C). This difference was not seen in fibroblasts with high and low concentrations of mesenchymal EVs (Figure S5D). To corroborate this and obtain quantitative data, fibroblasts were conditioned with fluorescently labelled (DiD, far red) CRC EVs, stimulated with TGF‐β and then assessed for immunoexpression of α‐SMA by flow cytometry (Figure S6). Fibroblasts which internalised mesenchymal (SW480) EVs demonstrated up to 10‐fold increase in α‐SMA expression upon TGF‐β treatment, independent of low‐ or high‐EV uptake. This stark increase was not observed in fibroblasts conditioned with epithelial (DLD1) EVs, although a much smaller (two‐fold) upregulation was still observed in both EV‐low and EV‐high populations (Figure S6). Taken together, these data suggest that uptake of miR‐200‐rich epithelial CRC EVs by fibroblasts abrogates α‐SMA upregulation at both RNA and protein levels.

Among many transcripts, the prime target of the miR‐200 family is *ZEB1*. The 3′‐UTR of *ZEB1* contains at least eight miR‐200 binding sites (Gregory et al., 2008). However, nearly all studies investigating post‐transcriptional regulation of *ZEB1* by miR‐200 were performed in epithelial cells (Gregory et al., 2008; Park et al., 2008). To identify whether miR‐200 plays a role in *ZEB1* regulation in our system, we preformed luciferase reporter assays in fibroblasts. Our results suggest that miR‐200 binding sites are functional and critical in the 3′‐UTR of *ZEB1* in the fibroblast setting (Figure S7).

To confirm that myofibroblast differentiation was regulated by the miR‐200/ZEB1 axis, fibroblasts were transfected with miR‐200 mimics (Figure 6b, Figure S8A) or *ZEB1* siRNA (Figure 6c, Figure S8B), then stimulated with TGF‐β. Both strategies decreased ZEB1 induction upon TGF‐β stimulation. In line with the involvement of ZEB1 in the TGF‐β pathway, activation of myofibroblast markers such as α‐SMA and fibronectin was also diminished (Figure 6b, c, Figure S8). Unlike with EV‐conditioning (Figure 6a), targeting ZEB1 directly by transfection of miR‐200 mimics or *ZEB1* siRNA reduced baseline as well as TGF‐β‐induced ZEB1 protein abundance (Figure 6b, c). In these instances, the basal and TGF‐β‐induced expression of ZEB1 target genes such as α‐SMA also decreases. This might suggest that the effect of EV‐contained miR‐200 is quantitatively less than that of transfected miR‐200 mimics or *ZEB1* siRNA but still enough to influence myofibroblast differentiation in the presence of TGF‐β.

Stress fibre formation is a hallmark of myofibroblast differentiation and α‐SMA is the main component of the myofibroblastic cytoskeletal structure (Hinz, 2007). Immunofluorescence experiments confirmed that *ZEB1* knockdown reduced α‐SMA accumulation and associated stress fibre formation upon TGF‐β treatment in both MRC5 and NCFs (Figure 6d). Therefore, ZEB1 induction through TGF‐β, which is a critical mediator of myofibroblast differentiation, can be regulated by miR‐200 transferred in EVs.

These results suggest that miR‐200 is contained in EVs derived from epithelial CRC cells, transferred to fibroblasts, leading to inhibition of the ZEB1‐dependent myofibroblast differentiation programme.

### 3.7 MiR‐200 predicts CAF profile in human CRC

**FIGURE 7:**
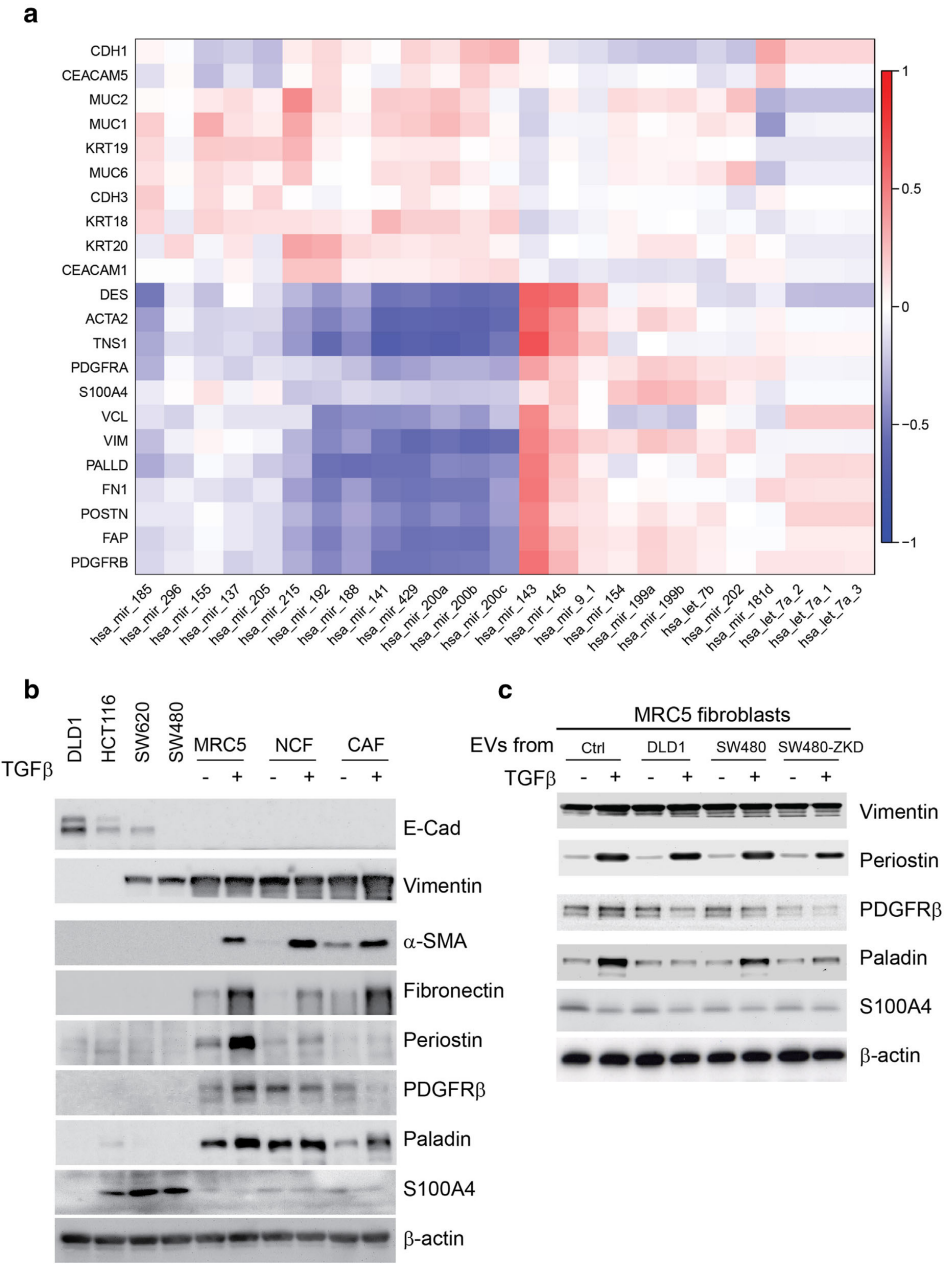
Correlation between EV‐miRNAs and CRC‐related epithelial and stromal genes. (a) Matrix constructed from 304 human CRC samples with matched miRNA and gene expression logCPMs. Unbiased hierarchical clustering of gene (mRNA) and miRNA expression according to correlation. Red to blue colour scale represents *r* from +1 to ‐1. (b) Protein expression by western blotting in CRC cells and fibroblasts (with or without TGF‐β) to demonstrate the expression of classical CAF markers across these cell types. (c) Protein expression by western blotting for additional CAF markers in MRC5 fibroblasts conditioned with DLD‐1 (epithelial), SW480 (mesenchymal) and SW480‐ZKD (epithelial) EVs, with or without TGF‐β stimulation. *E‐cadherin 130 kDa;* *vimentin 55 kDa;* *α‐* *SMA* *42 kDa;* *fibronectin 220 kDa;* *periostin 93 kDa;* *PDGFR* *β* *190 kDa;* *paladin 96 kDa;* *S100A4 12 kDa;* *β* *‐actin 42 kDa*. Lysates obtained to prepare Figure 6a were used in Figure 7c.

Myofibroblastic CAFs contribute to tumour progression (De Wever et al., 2008; Sahai et al., 2020; Tsujino et al., 2007). Having identified a critical relationship between miR‐200 and CAF phenotype *in vitro*, we looked to human CRC samples to see if this holds true. Three hundred and four CRC specimens with matched miRNA and gene expression data were identified in the TCGA dataset (Cancer Genome Atlas Research Network, et al. 2013). A signature of cancer cell‐associated and CAF‐associated genes was constructed according to the literature (Byrd & Bresalier, 2004; Lebleu & Kalluri, 2018; Majumdar et al., 2012; Shiga et al., 2015). The expression levels of the identified genes were correlated with the highest ranked EV miRNAs (by epithelial to mesenchymal ratio) from our miRNA array (shown in Figure 3). Reflecting their inhibitory role in EMT, miR‐200 family members correlated positively with epithelial tumour markers (e.g. *CDH1, KRT18*; Figure 7a). Supporting our *in vitro* data was the strong inverse relationship between miR‐200 and CAF markers, including *ACTA2* (α‐SMA) and *FN1* (fibronectin; Figure 7a). Other miRNAs, although abundant in epithelial EVs, did not show such a strong negative correlation with CAF markers. This suggests specificity of miR‐200 in determining stromal phenotype in CRC. However, we cannot exclude the possibility that there may be other miRNAs which correlate inversely with CAF markers because we limited the correlations to the miRNAs contained in our array. Taken with our *in vitro* findings, these results suggest that CRC tumours containing epithelial (well differentiated) CRC cells in abundance, secrete miR‐200‐rich EVs, which in turn inhibit TGF‐β‐driven differentiation of myofibroblasts. On the other hand, EVs secreted by mesenchymal (de‐differentiated) CRC cells, contain less miR‐200 and therefore allow TGF‐β‐driven myofibroblast formation.

It is well known that EMT induces mesenchymal gene expression in cancer cells (Lamouille et al., 2014). Since fibroblasts are derived from the mesoderm, it is only logical that there will be an overlap in the expression of genes we consider ‘CAF markers’ in fibroblasts and mesenchymal CRC cells. Therefore, we intended to dissect the data presented in Figure 7a, which were derived from bulk RNA sequencing of tumour tissue. We used CRC cell lines of defined EMT status in parallel with an established fibroblast line (MRC5) and a paired NCF and CAF obtained from the same patient (Bhome et al., 2017). The abundance of certain classical CAF markers (e.g. α‐SMA, fibronectin, periostin, PDGFRβ and paladin) was negligible in CRC cells (epithelial or mesenchymal) in comparison with fibroblasts and (TGF‐β‐stimulated) myofibroblasts (Figure 7b), suggesting that the contribution of CRC cells to the whole tumour expression of these markers is also negligible. Interestingly, S100A4 was expressed by certain CRC cells to a greater extent than fibroblasts, suggesting that it may mark de‐differentiation status of CRC rather than the CAF phenotype. Vimentin was expressed in both mesenchymal CRC cells and fibroblasts/myofibroblasts so it cannot be used as a CAF or fibroblast marker. Therefore, the majority of CAF markers presented in Figure 7a (barring S100A4 and vimentin) genuinely defined fibroblasts and the negative association of these markers with miR‐200 expression is incumbent on fibroblasts rather than cancer cells in CRC tissue.

We further investigated the effect of CRC EVs on the expression of these additional CAF markers in fibroblasts. We conditioned fibroblasts with epithelial and mesenchymal CRC EVs and induced myofibroblast differentiation with TGF‐β, as previously described. Our results suggest a differential effect of epithelial and mesenchymal EVs on paladin expression, similar to that observed for α‐SMA and fibronectin (Figure 7c) but this was not observed with other CAF markers. Therefore, we can confidently propose *ACTA2* (α‐SMA), *FN1* (fibronectin) and *PLLD* (paladin) as myofibroblast markers that are regulated in a similar fashion in the presence of epithelial or mesenchymal EVs. CRC EVs had a variety of different effects on other CAF markers (e.g., PDGFRβ or periostin), suggesting that they may play a role in the formation of different (non‐myofibroblastic) CAF subtypes in tumour stroma.

### 3.8 CRC EMT status determines fibroblast phenotype *in vivo*

**FIGURE 8:**
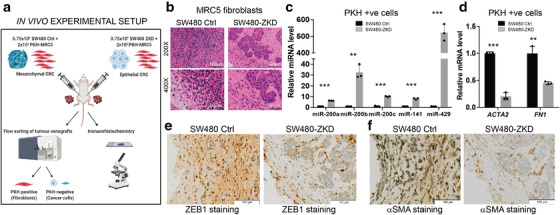
The role of CRC EMT in determining fibroblast phenotype *in vivo*. (a) Scheme of *in vivo* experimental setup. (b) Haematoxylin and eosin staining of sections from SW480 control and SW480‐ ZKD CRC xenografts. Scale bars represent 100 μm (200×; top panels) and 50 μm (400x; bottom panels). Representative of three xenografts from three animals. (c) MiR‐200 levels and (d) *ACTA2* (α‐SMA) and *FN1* (fibronectin) mRNA levels in PKH+ve cells (fibroblasts) from SW480 control and ZKD xenografts. Normalised to SW480 control xenografts, which were assigned the value 1. Statistical significance was determined by two‐tailed unpaired *t*‐test (**p < 0.01; ***p < 0.001). Values plotted are the means of three technical replicates from nine pooled xenografts. (e) ZEB1 and (f) α‐SMA immunohistochemical staining of sections from SW480 control and SW480‐ZKD xenografts. Scale bar represents 100 μm. Representative of three xenografts.

Encouraged by these data, we next sought to confirm this relationship using a controlled *in vivo* system. Isogenic but morphologically different CRC cells, representing mesenchymal (SW480 control) or epithelial (SW480‐ZKD) CRC variants, were co‐injected with PKH‐labelled fibroblasts into nude mice (six animals per group with two tumours per animal; *n* = 12). Tumours were excised after 14 days, subjected to histological assessment (*n* = 3) or disaggregated into single cells, pooled and flow‐sorted according to PKH‐positivity (*n* = 9), as illustrated in Figure 8a. Cell sorting data showed that in mesenchymal tumours (formed by SW480 control cells), 59.7% of viable single cells were injected fibroblasts and 13.3% were CRC cells. In epithelial tumours (formed by SW480‐ZKD cells), these proportions were 58.6% and 12.7%, respectively. Mesenchymal CRC cells were widely dispersed and uniformly mixed with fibroblasts (Figure 8b) creating diffuse, flat and scar‐like tumours. On the other hand, epithelial CRC cells formed visible epithelial islands (Figure 8b) representing differentiated CRC tumours, where cancer cells and fibroblasts were well‐segregated, appearing macroscopically as spherical nodules. We then sorted cells from these tumours, into PKH+ve (fibroblasts) and PKH‐ve (cancer cells). CRC cells from epithelial tumours contained more miR‐200a/c and ‐141 compared to those from mesenchymal tumours (Figure S9A), corresponding with their *in vitro* miRNA profiles (Figure 4c). Fibroblasts from epithelial tumours were more abundant in miR‐200a/b/c and ‐141 compared to those from mesenchymal tumours (Figure 8c). The increase in miR‐200 levels in CRC cells was associated with a reciprocal decrease in α‐SMA (*ACTA2*) and Fibronectin (*FN1*) mRNA levels in fibroblasts (Figure 8d). Immunohistochemical staining showed characteristic nuclear ZEB1 in mesenchymal CRC cells (SW480 control) and absence of ZEB1 in epithelial CRC cells (SW480‐ZKD; Figure 8e, Figure S9B), suggesting that our experimental model holds true *in vivo*. Furthermore, stromal α‐SMA staining was denser and less segregated in mesenchymal compared to epithelial tumours (Figure 8f, Figure S9C), suggesting that epithelial CRC cells inhibit α‐SMA+ve fibroblast formation.

Overall, when taken as a whole, these data demonstrate that in epithelial (differentiated) tumours, CRC cells convey miR‐200 to fibroblasts via EVs, inhibiting myofibroblast accumulation in the presence of TGF‐β. In comparison, in mesenchymal (de‐differentiated) tumours, CRC cells export less miR‐200 to fibroblasts, creating a permissive environment, which allows unrepressed TGF‐β‐driven myofibroblast differentiation through ZEB1 activation. However, we accept the fact that we did not manipulate EV production and show the direct involvement of EVs in myofibroblast differentiation *in vivo*. Therefore, we cannot exclude the possibility that non EV‐based factors are also contributing to this process.

## 4 DISCUSSION

Here, for the first time, we show that EV‐encapsulated miR‐200 mediates tumour‐stroma crosstalk in CRC, by regulating the fibroblast to myofibroblast switch. At a time when different CAF phenotypes are being discovered and characterised by single cell techniques, these data demonstrate a mechanism for what is being observed (Costa et al., 2018; Li et al., 2017). Most importantly, we provide an explanation for the accumulation of myofibroblastic CAFs in mesenchymal (CMS4) tumours, which have the worst prognosis (Guinney et al., 2015). We propose that epithelial CRC cells deliver miR‐200 to fibroblasts in EVs, attenuating stromal ZEB1 and decreasing sensitivity to TGF‐β‐mediated myofibroblastic differentiation. Conversely, mesenchymal (metastatic) CRC cells transfer less miR‐200 to fibroblasts, allowing unchecked response to TGF‐β, which results in fibroblasts becoming myofibroblasts.

Importantly, our data help contextualise recent studies which identify CAF heterogeneity in solid tumours (Costa et al., 2018; Li et al., 2017). In these exemplar studies, single cell techniques have been employed to identify different fibroblast populations in breast (Costa et al., 2018) and colorectal (Li et al., 2017) cancers. Costa et al. used multicolour flow cytometry to identify four breast fibroblast subtypes (S1‐S4), only two of which (S1 and S4) were myofibroblastic (α‐SMA ^high^) (Costa et al., 2018). Li et al. used single cell RNA sequencing to identify two fibroblast subtypes in colorectal tumours (CAF‐A and ‐B), of which only CAF‐B was myofibroblastic (Li et al., 2017). Interestingly, in both studies, the classical myofibroblastic marker (α‐SMA) differentiated intra‐tumoural (cancer‐associated) fibroblasts from juxta‐tumoural (nearby normal) fibroblasts, reinforcing the importance of the myofibroblastic phenotype in CAF biology. These observational studies highlighted stromal heterogeneity in solid tumours and demonstrated an association of CAF subtypes with non‐stromal cells (e.g., immune cells), which made us ask how different CAF subtypes come to exist in the first place. Here, we propose a mechanism which may explain this phenomenon, specifically how myofibroblastic differentiation of CAFs is controlled by neighbouring tumour cells. Nonetheless, questions remain regarding the contribution of EVs to CAF function and the formation of CAFs with different (non‐myofibroblastic) expression profiles (e.g., immune‐suppressive).

When considering the influence of cancer cell EMT on CAF subtypes, we must consider the effect of EMT‐inducing TFs on stromal cells. There is a large body of information regarding the role of these TFs in epithelial cells but comparatively little is known about their roles in fibroblasts. Nonetheless, it is becoming more apparent that the expression of EMT TFs is associated with the presence of CAFs (Baulida, 2017). Franci and colleagues showed that in cervical and colonic carcinomas, SNAIL expression was predominant in fibroblasts in close proximity to tumour cells, which were also SNAIL positive (Francí et al., 2006). Baulida's group demonstrated mechanical coupling in CAFs through a Snail1/RhoA/α‐SMA axis, with loss of SNAIL reducing the ability of TGF‐β to activate this pathway (Stanisavljevic et al., 2015). Sung et al. demonstrated co‐staining of Twist1 and FSP‐1 in gastric cancer stroma, suggesting an association between Twist and CAF activation, which was also associated with increased tumour invasion and metastasis (Sung et al., 2011). The importance of ZEB1 was highlighted by Bronsert and colleagues, who showed that stromal ZEB1 was an independent marker of prognosis in patients who have undergone resection for pancreatic ductal adenocarcinoma (Bronsert et al., 2014). Furthermore, Chang et al. showed that ZEB1 binds the *ACTA2* (α‐SMA) promoter, increasing its expression, resulting in a myofibroblastic phenotype (Chang et al., 2014). Our results support these findings and mechanistically explain why more (α‐SMA+ve) myofibroblasts are present in carcinomas with greater metastatic potential.

ZEB1 is regulated by the miR‐200 family of miRNAs (Gregory et al., 2008). However, the majority of studies showing this relationship have focused on cells of epithelial origin, including carcinoma cells (Bracken et al., 2008; Gregory et al., 2008; Park et al., 2008). In the present study, the miR‐200/ZEB1 axis was elucidated in fibroblasts. Tang et al. previously showed the importance of miR‐200 in breast cancer stroma (Tang et al., 2016). In their study, primary normal fibroblasts and CAFs were profiled for miR‐200. Normal fibroblasts consistently showed more miR‐200a/b/c, ‐141 and ‐429 than CAFs; however, the mechanism for this was not a focus of their study. Furthermore, overexpression of miR‐200 in CAFs resulted in a decrease in α‐SMA. Conversely, knockdown of miR‐200 in normal fibroblasts resulted in increased α‐SMA levels. Similarly, Yang and colleagues showed that miR‐200 negatively regulates activation of myofibroblasts in pulmonary fibrosis (Yang et al., 2012). In another recent study, miR‐200a/141 was shown to target *CXCL12β*, allowing the formation of a non‐immunogenic CAF subset in ovarian cancer (Givel et al., 2018). The relevance to our work was that miR‐200 levels were inversely associated with CAF accumulation. Therefore, several studies have identified a role for miR‐200 in determining fibroblast phenotype. However, none thus far have addressed the source of miR‐200 in fibroblasts (seeing as cells of mesenchymal lineage have little or no baseline miR‐200 (Park et al., 2008)), or how miR‐200 levels are upregulated. In this regard, our data show that cancer cell‐derived EVs can determine flux of miR‐200 in stromal fibroblasts.

One approach we used to further dissect the origin of miR‐200 in fibroblasts was to transfect miR‐200 inhibitors (antagomiRs) into epithelial CRC cells and follow miR‐200 abundance in EVs and recipient fibroblasts (Figure S4A‐C). Our results suggested that antagomiRs effectively reduce cellular, EV‐contained and transferred miR‐200 levels. Therefore, the reduction in miR‐200 in donor cells was mirrored in recipient fibroblasts. However, there are uncertainties related to whether antagomiRs: (i) bind to pri‐miR and inhibit the formation of mature miRNAs, (ii) bind to mature miRNAs and make them unavailable for their function, or (iii) bind to miRNAs residing in RISC complexes and allow their disassociation (Stenvang et al., 2012). Of these three possibilities, the second and third are more widely accepted; however, this does not explain changes in endogenous miRNA levels upon antagomiR transfection, as we observed with miR‐200 (Figure S4A). Importantly, reduction of endogenous miRNA levels with antagomiRs has been observed by other groups and for other miRNAs, such as miR‐16, both *in vitro* and *in vivo* (Krützfeldt et al., 2007). Another aspect to consider is that antagomiRs can have off‐target effects by binding to unintended miRNAs or interfering with miRNA processing machinery (Stenvang et al., 2012). Therefore, we did not perform any functional studies with antagomiRs.

Interestingly, there are several parallels between our work and a recent study on the development of pulmonary fibrosis (Yao et al., 2019). Here, alveolar type II (ATII) cells were shown to influence fibroblast phenotype. Conditioned media from mesenchymal ATII cells allowed uninhibited myofibroblast differentiation in MRC5 fibroblasts, which was attenuated in the presence of conditioned media from epithelial ATII cells. Furthermore, in sections from fibrotic lungs, ZEB1 expression was identified in fibroblastic foci adjacent to ZEB1‐positive alveolar epithelial cells, suggesting that ZEB1 is critical in the crosstalk between epithelial cells and fibroblasts. Clearly, there are several similarities between this study and our work. In both cases, it was shown that the epithelial compartment (lung parenchyma or cancer cells) can alter phenotype of stromal cells, through paracrine mechanisms, and that this is determined by EMT status of the epithelial cells in that tissue. Furthermore, the importance of ZEB1 in regulating fibroblast phenotype was highlighted in both studies. However, despite providing an in‐depth analysis of the alveolar cell secretome, Yang et al. did not provide mechanistic insight into the regulation of ZEB1 in fibroblasts (Yao et al., 2019). In that context, EV‐derived miR‐200 may also be critical in fibrosis.

A TGF‐β‐driven model of myofibroblast differentiation was used in the present study because it is particularly relevant in CRC. Despite the majority of CRCs having deactivating TGF‐β pathway mutations (*TGFBR1, TGFBR2, SMAD4, SMAD2, SMAD3*), colorectal tumours produce significant amounts of TGF‐β, creating a TGF‐β rich environment, to which only the stromal compartment can respond (Calon et al., 2012). Importantly, we showed that EMT status does not affect TGF‐β production by CRC cells (Figure S1C), suggesting that differences in CAF phenotypes between epithelial and mesenchymal tumours (e.g. CMS2/3 vs. CMS4) are likely to be independent of local TGF‐β availability.

With respect to tumour‐stroma crosstalk, we highlighted EV‐encapsulated miRNA transfer as one mechanism but others may exist in parallel, such as juxtacrine signalling and secretion of soluble factors (Calvo et al., 2013; Labernadie et al., 2017). The *in vivo* finding, that stromal miR‐200 correlates with cancer cell miR‐200, strongly suggests that changes in miR‐200 in one compartment are transmitted to the other. To support this, our *in vitro* findings clearly demonstrate that CRC cells can transfer miR‐200 to fibroblasts. To show that the observed effects on fibroblast phenotype are EV‐dependent *in vivo* can be experimentally challenging. For example, knocking down Rab27 to reduce EV secretion may be considered an option. However, this approach may also have several EV‐independent effects on cancer cells (Bobrie et al., 2012). Similarly, the nMase‐2 inhibitor GW4869, which uncouples endosomal miRNA loading, may result in significant non‐specific effects in fibroblasts and cancer cells (Unal et al., 2017; Vuckovic et al., 2017). All these approaches may influence or limit empirical exploration of EV function, especially when using *in vivo* models. For these reasons, we are currently developing new technologies to manipulate EV production from cancer cells in a more controlled and temporal manner, without altering systemic physiology. Therefore, in future we will be in a position to test the *in vivo* involvement of EVs in myofibroblast differentiation and address this limitation.

Another experimental consideration is the use of TCGA data, which is derived from bulk RNA sequencing of whole colorectal tumours. Although, this clearly shows the negative association between miR‐200 and CAF markers, bulk sequencing does not allow us to distinguish expression in stromal and cancer cell compartments. To directly assess this, we would need to separate stromal and epithelial compartments in primary tumours by laser capture microdissection and quantify miR‐200 (epithelial) and activated fibroblast (stromal) markers. Further correlation of epithelial miR‐200 and stromal CAF markers with CMS subtype is also possible. However, this would require prospective bulk RNA sequencing of primary tumours to establish the CMS subtype and simultaneous laser microdissection. Either way, the result would only enable us to make further correlations, albeit between stromal or epithelial‐specific markers and CMS subtype. However, this would only marginally add mechanistic to future studies.

Overall, our study provides a mechanism for the accumulation of myofibroblastic stroma in CMS4 tumours, pointing a spotlight on the miR‐200/ZEB1 axis in fibroblasts, and its regulation by tumour cell‐derived EVs. The demonstration that EMT status of CRC determines fibroblast phenotype is a critical link in understanding how different fibroblast phenotypes come to exist in tumours. This work is especially timely because it provides mechanistic insight into recently published observations of stromal heterogeneity in solid cancers. A better understanding of this concept will enable development of prognostic stromal biomarkers and the ability to modulate CAF phenotypes for therapeutic advantage.

## CONFLICT OF INTEREST

The authors declare no competing interests.

## AUTHOR CONTRIBUTIONS

The project was conceptualised by RB, AHM and AES. RB acquired all data, with support from ME (NCF experiments), VJ (5EU labelling/ transfer of miRNAs), LMH (miRNA array) and MM (immunocytochemistry). JT provided support with EV isolation and conducted NTA in ODW's laboratory. RB, AHM and AES analysed and interpreted the data. RB, AHM and AES wrote the manuscript with substantial revisions from JNP, GJT and ODW. All authors approved the article in its final form.

## Supporting information

Supplementary information

## Data Availability

ExomiR data have been deposited at Exocarta (http://www.exocarta.org/) and will be available concomitant with publication. Other data that support the findings are available upon reasonable request from the corresponding authors.
