# Supplementary material for: Epithelial to mesenchymal transition influences fibroblast phenotype in colorectal cancer by altering miR‐200 levels in extracellular vesicles
Source: J Extracell Vesicles. 2022 May 20;11(5):e12226. doi: 10.1002/jev2.12226 (PMC9122835; doi:10.1002/jev2.12226)
Supplement: Supplementary file 1 — Supplementary information [file JEV2-11-e12226-s001.pdf]

SUPPLEMENTARY MATERIAL

Epithelial to mesenchymal transition influences fibroblast phenotype in colorectal cancer by altering miR-200 levels in extracellular vesicles

Rahul Bhome[1,2], Muhammad Emaduddin[1], Victoria James[3], Louise M House[1], Stephen M Thirdborough[1], Massimiliano Mellone[1], Joeri Tulkens[4], John N Primrose[2], Gareth J Thomas[1], Olivier De Wever[4], Alex H Mirnezami[1,2*], A Emre Sayan[1*]

[1] Cancer Sciences Unit, University of Southampton, Southampton, UK

[2] University Surgery, University of Southampton, Southampton, UK

[3] School of Veterinary Medicine and Science, University of Nottingham, UK

[4] Laboratory of Experimental Cancer Research, Department of Human Structure and Repair, Ghent University, Belgium

Materials & Correspondence:

Dr AE Sayan, Cancer Sciences, University of Southampton, Somers Building, Southampton General Hospital, Southampton, SO16 6YD, UK. Email: a.e.sayan@soton.ac.uk

Professor AH Mirnezami, Cancer Sciences, University of Southampton, Somers Building, Southampton General Hospital, Southampton, SO16 6YD, UK. Email: a.h.mirnezami@soton.ac.uk

Supplementary Figures: S1-S9

Supplementary Methods

Supplementary References

SUPPLEMENTARY FIGURE 1

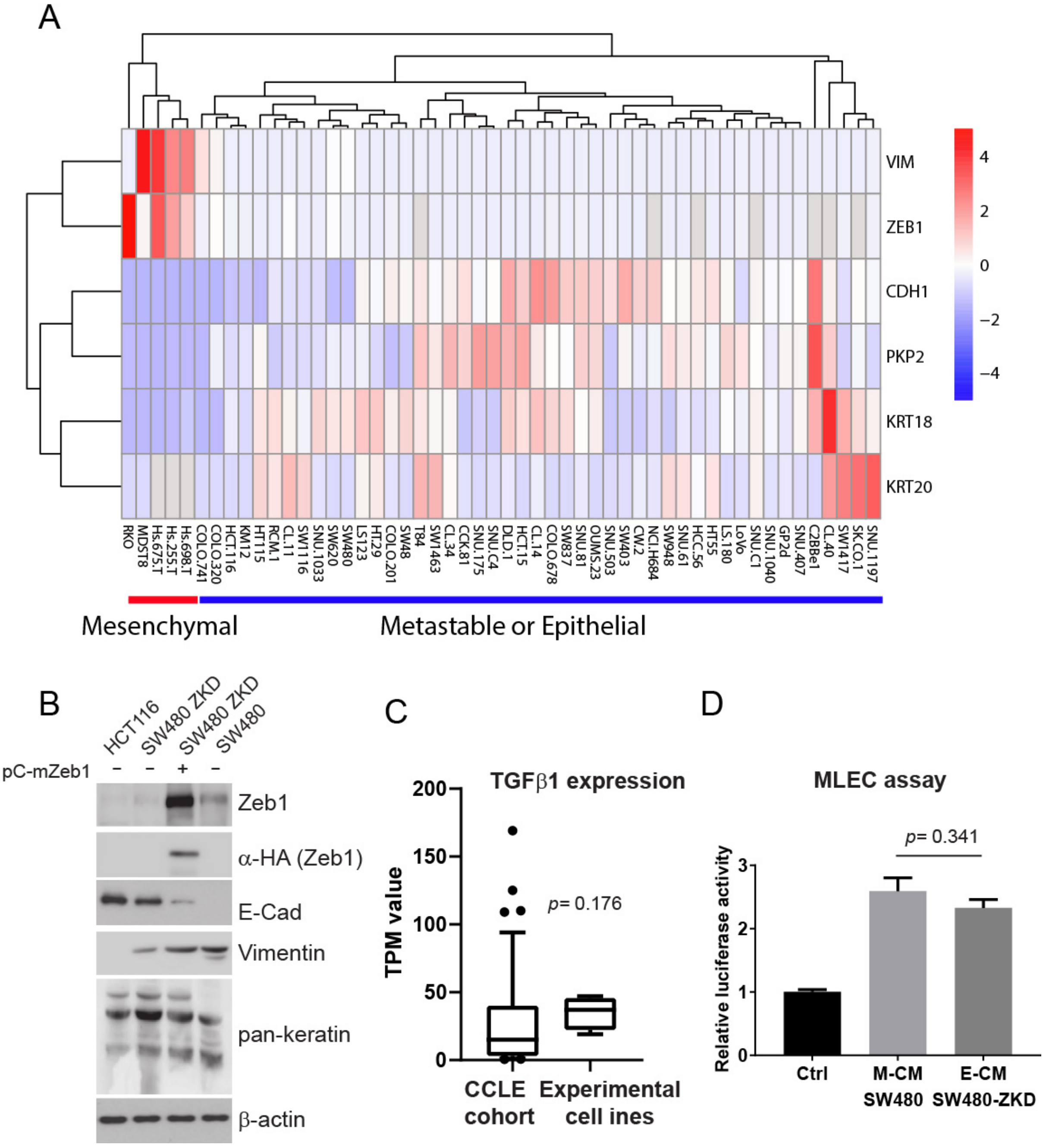

Figure S1. EMT marker and TGF-β expression in CRC cell line models. (**A**) Gene expression of EMT markers for 52 CRC cell lines in the CCLE dataset. Logarithmic scale (transcripts per million). (**B**) Protein expression of EMT markers in SW480-ZKD cells transfected with murine *zeb1,* to demonstrate rescue of the phenotype. HCT116 and SW480 cells were used as controls showing epithelial and mesenchymal expression patterns, respectively. Exogenous murine Zeb1 protein was detected using HA-tag antibody. (**C**) *TGFB1* expression in the 51 CCLE CRC cell lines compared to

selected experimental CRC cell lines (DLD-1, HCT116, SW620, SW480). Box plots show median (10-90th percentiles), range and outliers. Statistical significance was determined by two-tailed Mann-Whitney U Test (U value 59.5). *TPM – transcripts per million.* (**D**) MLEC assay. Conditioned medium from SW480 control cells (mesenchymal; *M-CM*) and ZKD cells (epithelial; *E-CM*) assayed for TGF-β content by exposure to MLEC cells. Luciferase activity was normalised to untreated (Ctrl) cells, which were assigned the value 1. Statistical significance was determined by two-tailed unpaired t-test.

SUPPLEMENTARY FIGURE 2

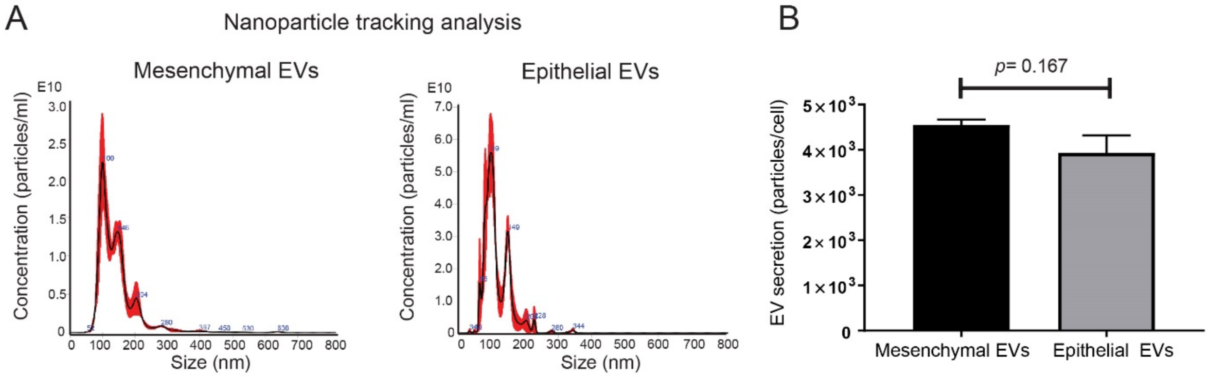

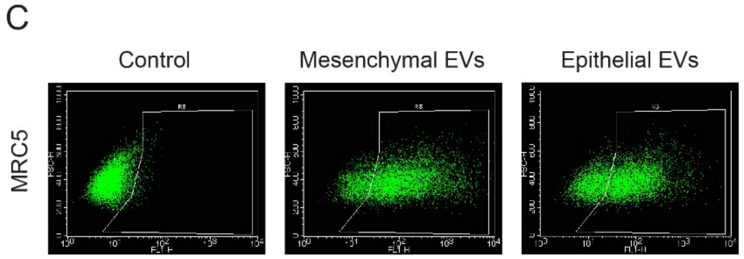

%	Control	M-EVs	E-EVs
negative	96.4	18.1	16.9
positive	3.6	81.9	83.1
	C vs M	p<0.0001	
		C vs E	p<0.0001
		M vs E	p=1.0

Figure S2. Comparison of mesenchymal and epithelial CRC EVs. (**A**) Nanoparticle tracking analysis of SW480 control (mesenchymal) and SW480 ZKD (epithelial) EVs from five videos, each 90 s duration. (**B**) EV production in particles per cell (cell count immediately prior to EV isolation) for SW480 control and ZKD cells (five separate experiments). Statistical significance determined by two-tailed unpaired t-test. (**C**) Uptake of DiO-labelled SW480 control (mesenchymal - *M*) and SW480 ZKD (epithelial - *E*) EVs by MRC5 fibroblasts. Control represents fibroblasts conditioned with DiO-labelled medium. Representative of three experiments. Statistical significance determined by two-tailed Fisher's exact test.

SUPPLEMENTARY FIGURE 3

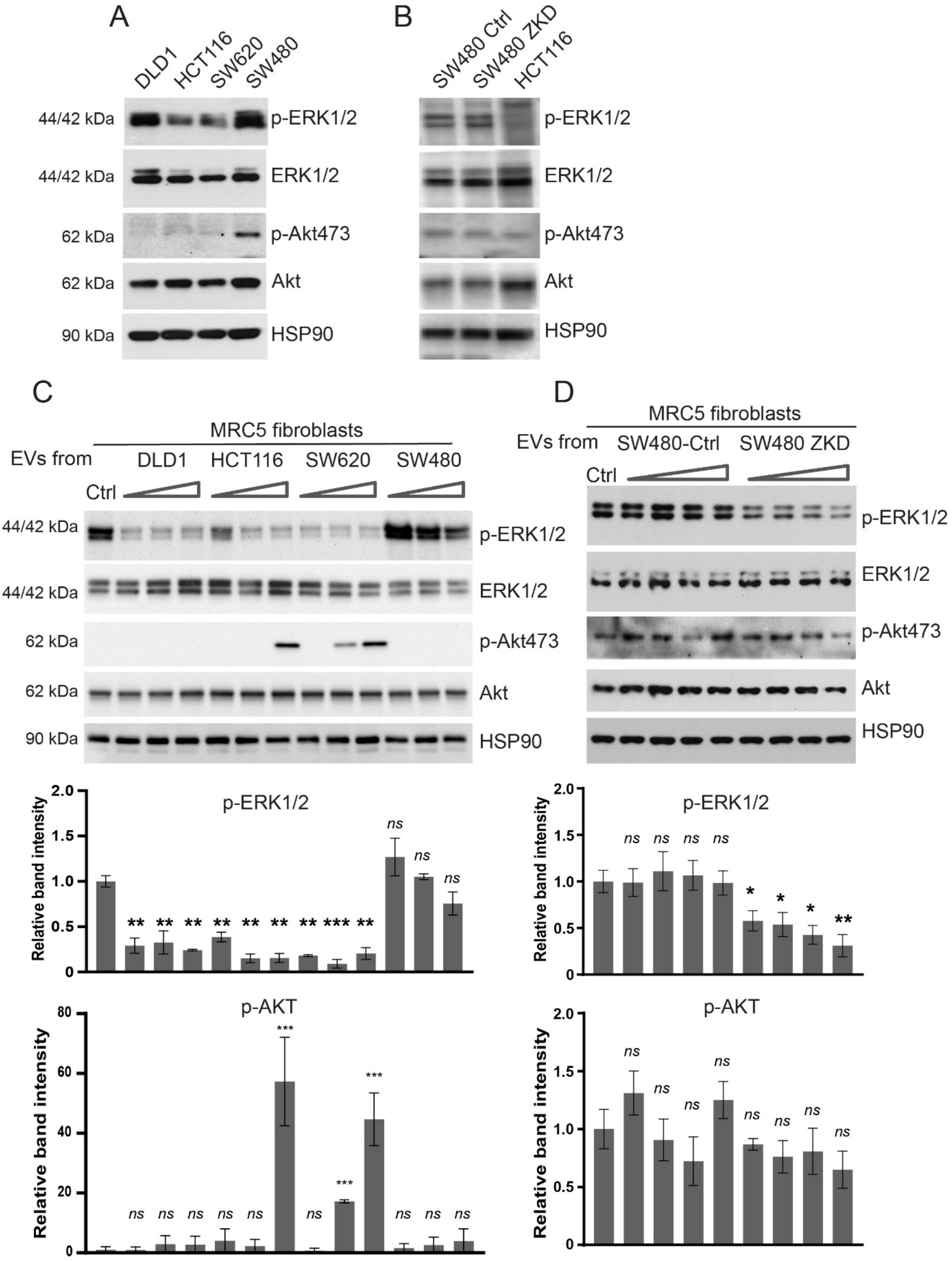

Figure S3. Effect of epithelial and mesenchymal CRC EVs on fibroblast signalling pathways.

Baseline ERK and AKT activity in (**A**) CRC cells and (**B**) SW480 control and SW480-ZKD cells

(MET model). (**C**) Total and phosphorylated ERK and AKT expression in MRC5 fibroblasts conditioned with EVs from CRC cells at increasing concentrations (0.5, 1.5 and 4.5 x10^9 particles/ml) for 24h. EV-depleted medium was used as a control. (**D**) Conditioning of MRC5 cells with EVs from SW480 control and SW480-ZKD cells (0.5, 1, 1.5 and 4.5 x10^9 particles/ml) for 24h. HSP90 was used as an equal loading control. Representative of three experiments. For (C) and (D), band intensities for p-ERK1/2 and p-Akt473 were normalised to HSP90 and then to total ERK1/2 or total Akt, with the first band assigned the value 1. Values plotted are from three independent experiments. Statistical significance (each band compared to the first band) was determined by two-tailed unpaired t-test (*ns* – not significant; * p<0.05; ** p<0.01; *** p<0.001).

SUPPLEMENTARY FIGURE 4

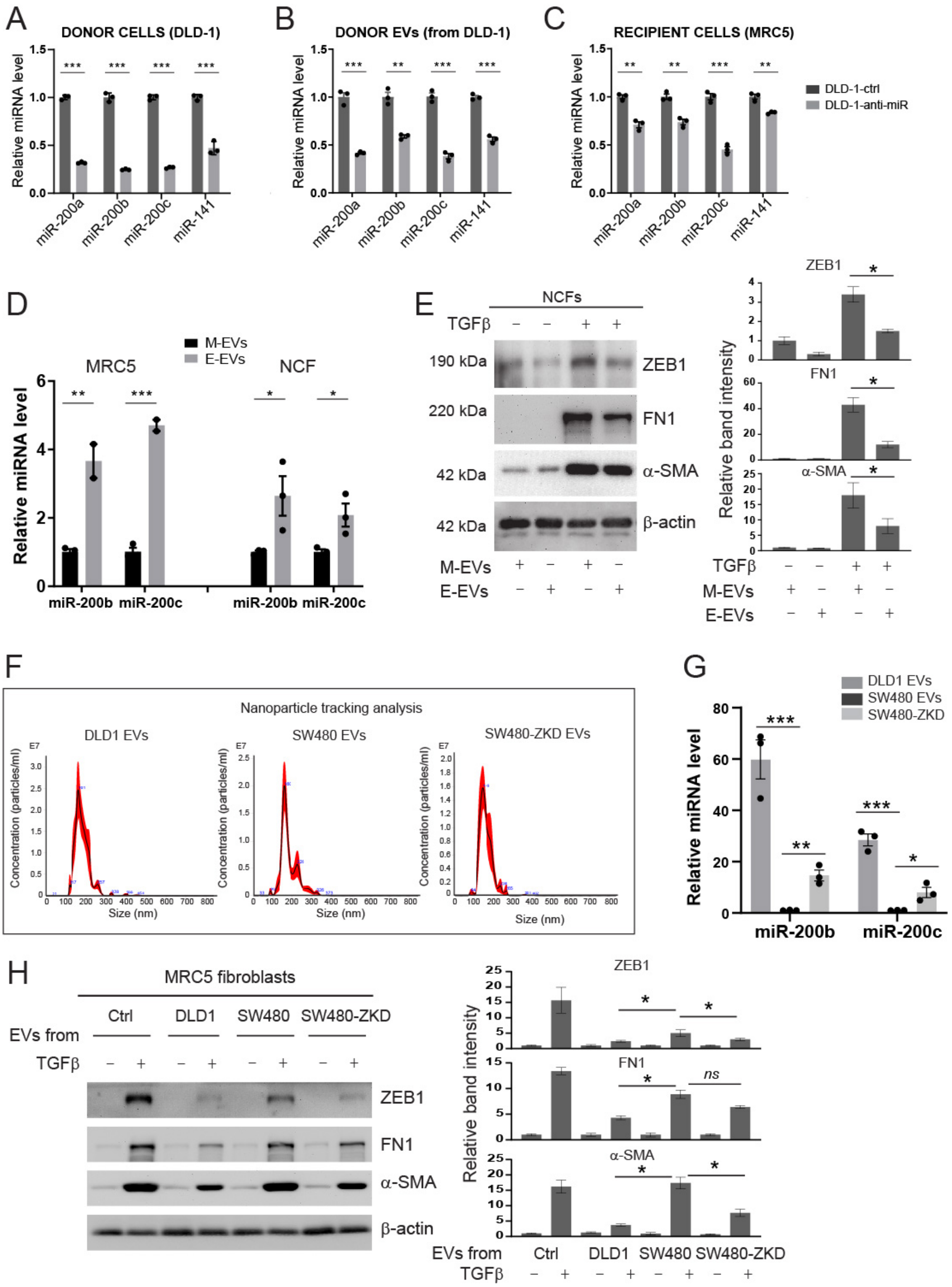

Figure S4. Effects of epithelial and mesenchymal CRC-EVs on myofibroblast differentiation. MiR-200 levels in (**A**) DLD-1 control and antagomiR-transfected (miR-200 inhibitor) cells, (**B**) EVs and

(**C**) MRC5 fibroblasts conditioned with EVs. MiRNA levels were normalised to control cells, EVs, or fibroblasts conditioned with control EVs, which were assigned the value 1. (**D**) MiR-200 levels in MRC5 fibroblasts and NCFs conditioned for five days with mesenchymal (SW480, *M*-) or epithelial (DLD-1, *E*-) EVs at a concentration of 1.5×10^9 EVs/ml (quantified using Nanosight). All levels normalised to M-EV conditioned fibroblasts, which was set to the value 1. (**E**) Protein expression of myofibroblast markers in NCFs conditioned with E- or M-EVs and stimulated with TGF-β. Band intensities of ZEB1, fibronectin and α-SMA are relative to β-actin and normalised to the first lane of the blot which was given the value 1. Values plotted are from three independent experiments. Statistical significance was determined by two-tailed unpaired t-test (*ns* – not significant; * $p<0.05$; ** $p<0.01$; *** $p<0.001$). (**F**) Nanoparticle tracking analysis of DLD1, SW480 and SW480-ZKD EVs, isolated using size exclusion chromatography, from five separate videos, each 90s duration. (**G**) MiR-200 levels in DLD1 (epithelial), SW480 (mesenchymal) and SW480-ZKD (epithelial) EVs isolated using size exclusion chromatography. MiRNA levels were normalised to DLD1 EVs, which were assigned the value 1. Values plotted are means of three technical replicates from three experiments. (**H**) Protein expression of myofibroblast markers by western blotting in MRC5 fibroblasts conditioned with DLD1 (epithelial), SW480 (mesenchymal) or SW480-ZKD (epithelial) EVs before and after TGF-β stimulation. Band intensities are relative to β-actin and normalised to the first lane of the blot which was given the value 1. Values plotted are from three independent experiments. Statistical significance was determined by two-tailed unpaired t-test (*ns* – not significant; * $p<0.05$; ** $p<0.01$; *** $p<0.001$).

SUPPLEMENTARY FIGURE 5

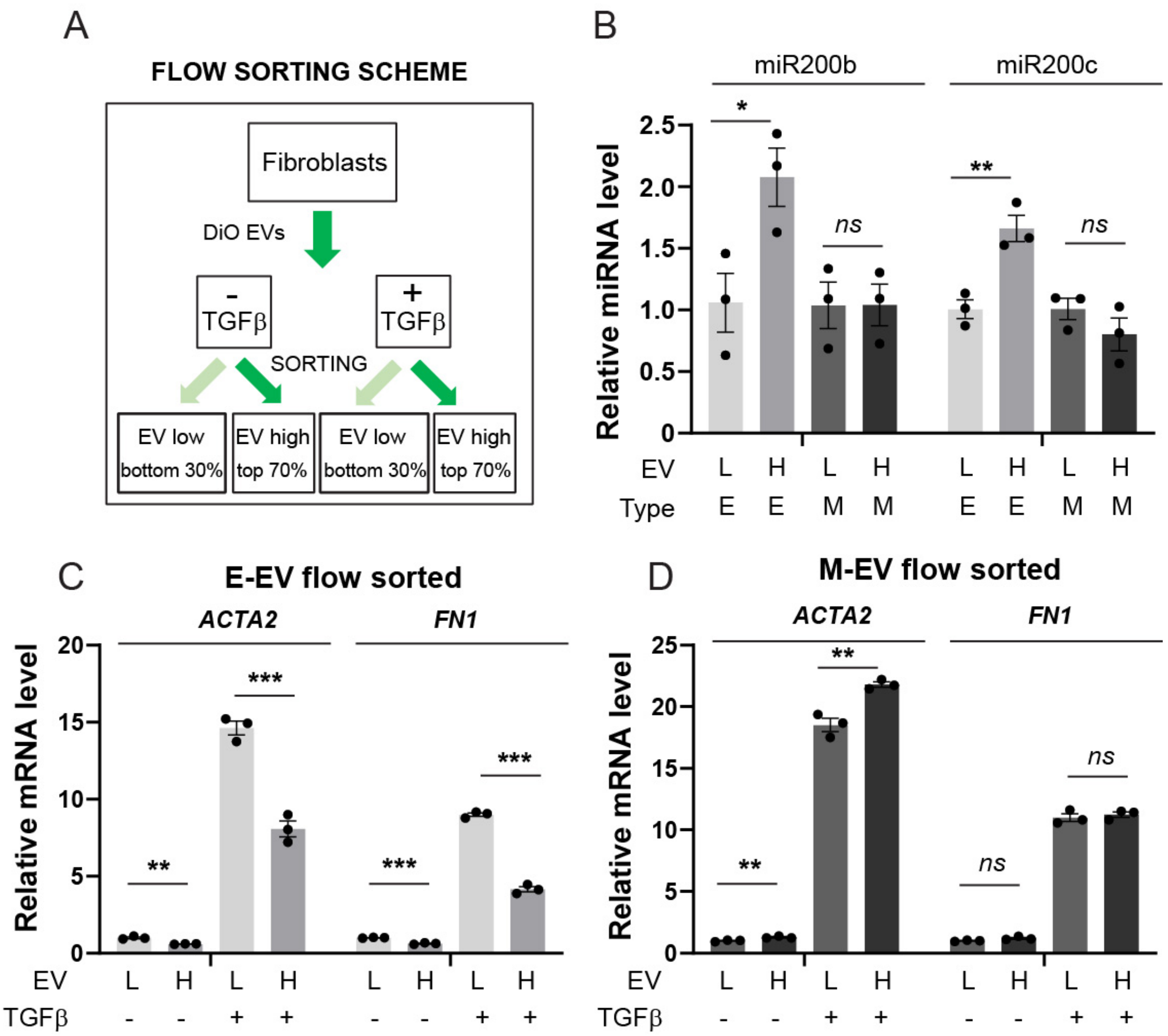

Figure S5. Sorting of EV-high and EV-low fibroblasts. (**A**) Schematic of experimental set up. (**B**) MiR-200 levels in fibroblasts (+/- TGF-β) with low (L; 0-30th centile) and high (H; 70-100th centile) concentrations of fluorescently labelled E-EVs (DLD1) and M-EVs (SW480). *ACTA2* and *FN1* levels in fibroblasts with low and high concentrations of (**C**) E-EVs, or, (**D**) M-EVs. MiRNA/ mRNA levels were normalised to EV^{low}/ TGF-β^{-} fibroblasts, which were assigned the value 1. Values plotted are means of three technical replicates from three experiments. Statistical significance was determined by two-tailed unpaired t-test (*ns* – not significant; * *p*<0.05; ** *p*<0.01; *** *p*<0.001).

SUPPLEMENTARY FIGURE 6

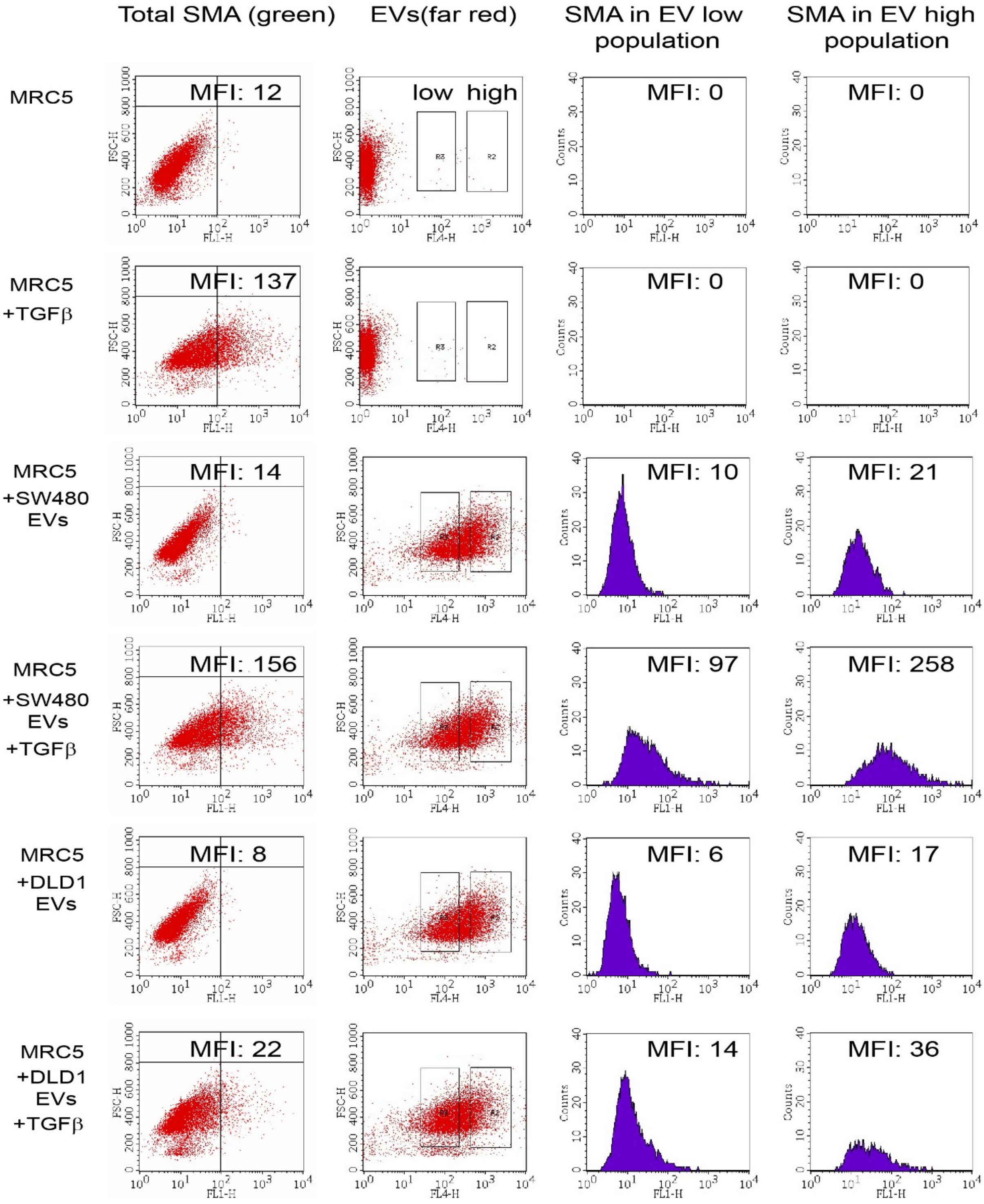

Figure S6. Immuno-detection of α-SMA in EV^{low} and EV^{high} fibroblasts/ myofibroblasts. MRC5 fibroblasts were conditioned with DiD-labelled (far red, FL4 channel) SW480 (mesenchymal) or DLD1 (epithelial) EVs as described previously for three days and treated with TGF-β for an additional two days to initiate myofibroblastic differentiation. Fibroblasts were then collected, fixed,

permeabilised and assayed for α-SMA immunoexpression. From left to right: total α-SMA, FL4 gating to identify cells with low and high EV uptake, α-SMA in EV^{low} and EV^{high} population. Mean fluorescence intensity (MFI) is indicated in each plot/ histogram.

SUPPLEMENTARY FIGURE 7

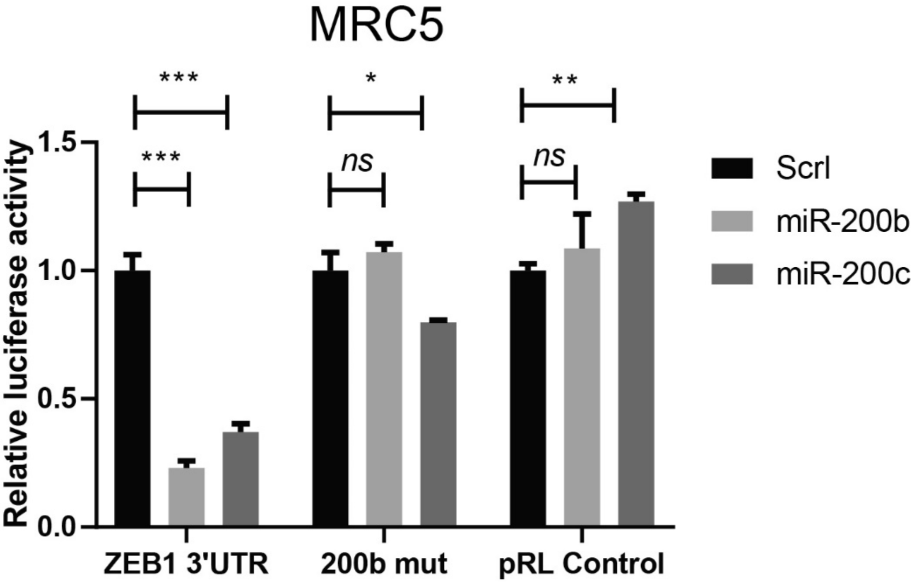

Figure S7. 3’UTR luciferase reporter assays for miR-200/*ZEB1* targeting in MRC5 fibroblasts. Cells were transfected with *ZEB1* 3’UTR, mutant 3’UTR or control 3’UTR, together with miR200b/c or scrambled control. Luciferase activity was normalised to scrambled control, which was assigned the value 1. Statistical significance was determined by two-tailed unpaired t-test (ns – not significant; * p<0.05; ** p<0.01; *** p<0.001). *Scrl - scrambled control; 200b mut - ZEB1 3’UTR mutated at all miR-200b binding sites; pRL control - control 3’UTR (fragmented HNF4A 3’-UTR).*

SUPPLEMENTARY FIGURE 8

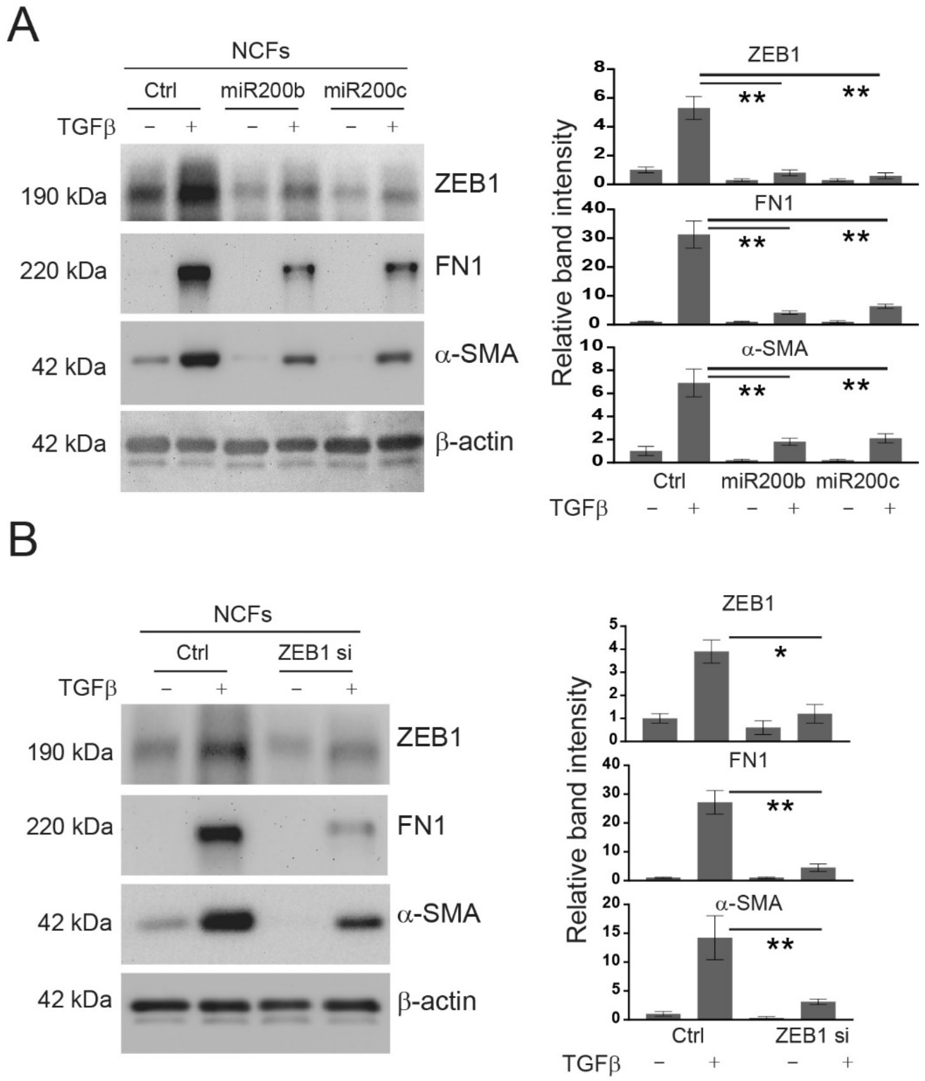

Figure S8. The effect of miR-200 overexpression or *ZEB1* downregulation on myofibroblast differentiation. Myofibroblast marker expression in NCFs (+/- TGF-β) transfected with (**A**) miR-200b or miR-200c, or, (**B**) ZEB1 siRNA. Band intensities in panels (A) and (B) for ZEB1, fibronectin and α-SMA are relative to β-actin and normalised to the first lane of the blot which was given the value 1. Values plotted are from three independent experiments.

SUPPLEMENTARY FIGURE 9

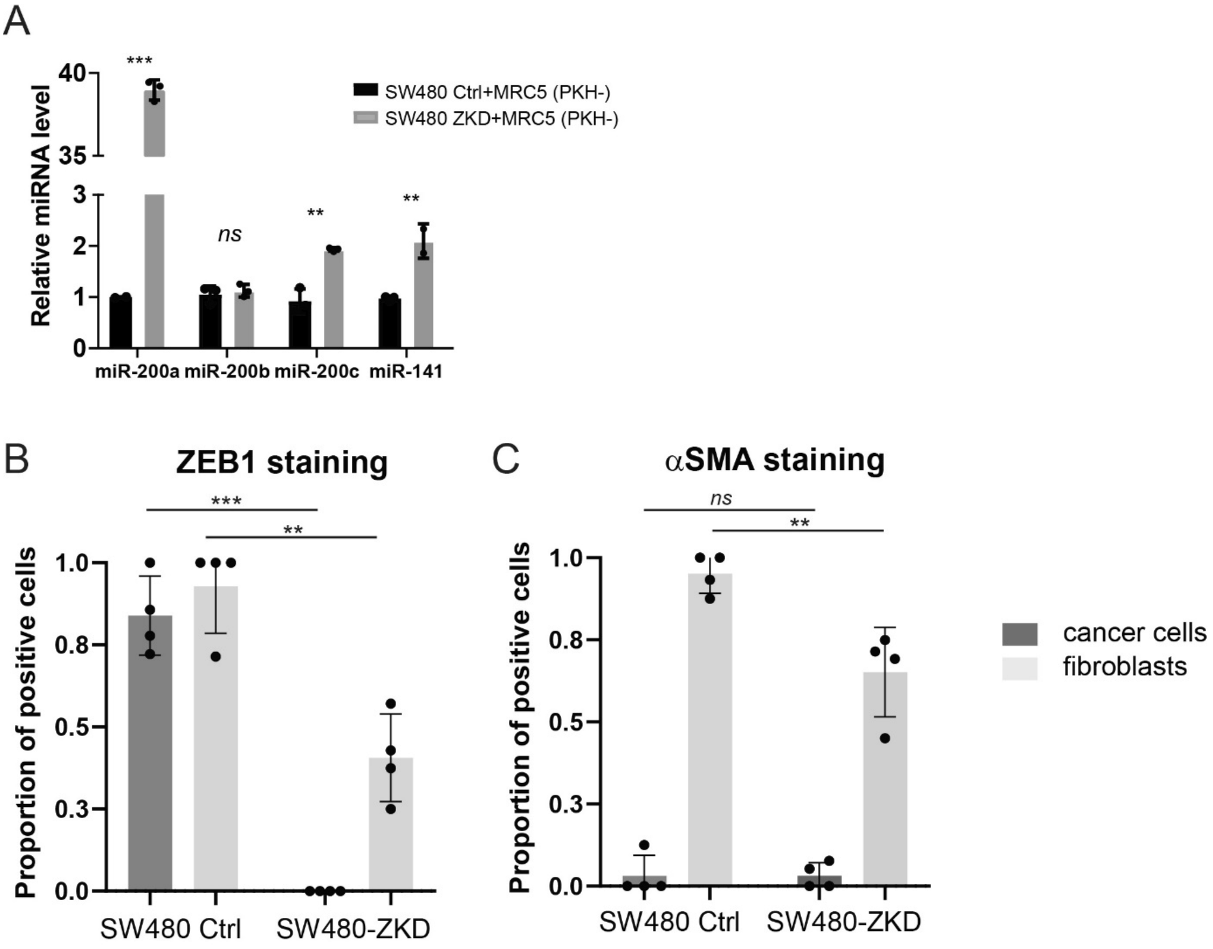

Figure S9. Supplementary *in vivo* data. **(A)** MiR-200 levels in PKH-negative (cancer) cells from CRC xenografts. MiRNA level was normalised to cells from SW480 control xenografts, which were assigned the value 1. Values plotted are means of three technical replicates from three xenografts. (**B**) ZEB1 and (**C**) α-SMA immunohistochemical staining quantification. Positivity was assessed by visually identifying the identity of the cell and assessing immunostaining qualitatively and quantitatively using a gridded screen with Optical Density (OD) calculator. Values plotted are the means of nine (100 μm^2) fields of view from three independent sections. Statistical significance was determined by two-tailed unpaired t-test (ns – not significant; ** p<0.01; *** p<0.001) for A-C.

SUPPLEMENTARY METHODS

Cell lines

DLD1, HCT116, SW620 and SW480 human colorectal adenocarcinoma cells were procured from American Type Culture Collection. MRC5 human foetal lung fibroblasts were procured from The European Collection of Authenticated Cell Cultures. Hepa1-6 cells (metastatic mouse hepatoma) were purchased from German Collection of Microorganisms and Cell Cultures. All commercially acquired cell lines were characterised by STR profiling (Eurofins Genomics, Luxembourg) every six months. NCFs were derived from biopsies of normal colon as described previously (CRA-463-14/ pair#2) (Bhome et al. 2017). MLEC cells, stably transfected with truncated *PAI-1* promoter fused to firefly luciferase, were used for the functional TGF-β assay. All cells were grown in DMEM, supplemented with 10% FBS (v/v), 2mM L-Glutamine (cat no: G7513; Sigma) and 1% penicillin-streptomycin (100 units penicillin – 100 μg streptomycin/ml, cat no: P4333; Sigma), and maintained at 37°C in a humidified atmosphere of 5% CO_2.

Cloning of mouse zeb1 cDNA and generation of SW480 ZEB1 knock down cells

Mouse *zeb1* cDNA was amplified from the Hepa1-6 cell line, cloned into pGEM3 cloning vector with an N-terminal HA-tag, and sequence verified. Next, it was sub-cloned into pCDNA3.1 vector (Invitrogen) using the SCLIC technique (Li and Elledge 2012). HEK293T cells were transfected with ViraPower packaging mix (cat no. K497500; ThermoFisher) and validated *ZEB1* pLKO shRNA (cat no. SHCLND-NM_030751; TRC no: TRCN0000364631 (CCTACCACTGGATGTAGTAAA), Sigma) or pLKO (control, Sigma, cat no: SHC001), using Lipofectamine 3000 (cat no. L3000008; ThermoFisher). Viral supernatant was collected at 48 and 96h post-transfection, filtered (0.22 μm filter) and stored at -80°C.

For generating ZEB1 knockdown cells, 3 x 10^5 SW480 cells were seeded into individual wells of a 6-well plate. The following day, medium was replaced with 1 ml viral supernatant (pLKO-Zeb1-shRNA or pLKO), containing 8 μg/ml hexadimethrine bromide (Polybrene; cat no. H9268; Sigma). Cells were

washed after 24h, trypsinised and cultured in the presence of puromycin (2 μg/ml) for 2 weeks. ZEB1 knock down was assessed by western blotting.

ZEB1 knockdown rescue

SW480-ZKD cells were seeded into 6-well plates at 40% confluency. The following day, cells were transfected with 3 μg of murine *zeb1* cDNA expression construct (pCDNA-mZeb1) or control plasmid (pCDNA3.1) using Lipofectamine 2000. Biochemical hallmarks of EMT were determined by western blotting for endogenous ZEB1, exogenous ZEB1 (α-HA7; 1:5000; Sigma), E-cadherin, vimentin and pan-keratin at 72h.

MLEC assay

5x10^5 MLEC cells were seeded into each well of a 24-well plate. The following day, cells were treated with 0, 250, 500, 1000 or 2000 pg/ml TGF-β (cat no. 240-B; R&D Systems) to generate a dose-response curve. In parallel, cells were treated with conditioned medium (diluted 1:1 with fresh medium) from SW480 control or SW480-ZKD cells. All standards and experimental conditions were conducted in triplicate. At 24h, medium was aspirated and replaced with 100 μl Passive Lysis Buffer (cat no. E1941; Promega). Cells were then incubated at room temperature for 20 min on a rocker. 20 μl of each lysate was then transferred to each well of a 96-well plate, and 100 μl Luciferase Assay Reagent (cat no. E1500; Promega) was added to each well. Firefly luciferase activity was then measured using a luminometric plate reader (Varioskan LUX Multimode Microplate Reader; ThermoFisher). In parallel, 5 μl of cell lysate was used to measure protein concentration. Firefly luciferase activity was normalised by protein concentration for each well.

Western blotting

Briefly, 20-30 μg of protein was separated under reducing conditions in 8, 10, 12 or 15% SDS-PAGE gels. Proteins were then transferred on to nitrocellulose membranes (Amersham Protan 0.1 NC nitrocellulose membranes (cat no. 10600000; GE Healthcare), blocked in 4% milk, and then incubated with primary antibodies. For EV characterisation: Alix (3A9; 1:500), TSG101 (4A10; 1:500; Abcam), CD63 (Ts63; 1:500), CD81 (1.3.3.22; 1:500; ThermoFisher), *β*-actin (C4; 1:5000; BD Biosciences) and

cytochrome C (4272; 1:1000; Cell Signalling Technology) were used. For EMT status: Zeb1 (H-102; 1:500; Santa-Cruz Biotechnology), E-cadherin (clone 36; 1:1000; BD Biosciences), pan-cytokeratin (C2562; 1:10 000; Sigma) and vimentin (D21H3; 1:1000; Cell Signalling Technology). For ERK/Akt pathways: p44/42 MAPK/ ERK1/2 (137F5; 1:2000), phospho-p44/42 MAPK /ERK1/2 (D13.14.4E; 1:1000), Akt (C67E7; 1:1000) and phospho-AKT (Ser473; D9E; 1:500; Cell Signaling Technology). For myofibroblast trans-differentiation: fibronectin-EDA (MAB1940; 1:2000; Merck Millipore; Burlington, USA), α-SMA (1A4; 1:2000; Sigma), vimentin (D21H3; 1:1000; Cell Signalling Technology), paladin (nbp1-80952; 1:500; Novus Biologicals), periostin (ab14041; 1:1000; Abcam) PDGFRβ (#3169, 1:1000; Cell Signalling) and S100A4 (07-2274; 1:500: Merck Millipore). β-actin (C4; 1:5000) and HSP90 (68/Hsp90; 1:1000; BD Biosciences) were used as equal loading controls. Membranes were then washed and incubated with horseradish peroxidase-conjugated secondary antibodies (polyclonal swine anti-rabbit (cat no. P021702-2) and polyclonal rabbit anti-mouse (cat no. P016102-2); 1:3000; Dako). Membranes were then washed again, and specific signal was visualised using SuperSignal West Dura (cat no. 34075; ThermoFisher) or West Femto (cat no. 34094; ThermoFisher) chemiluminescent detection kits. Where necessary, band intensity was quantified in Image J. Values shown are relative to β-actin/ HSP90 and normalised to the first lane of the blot, which was given the value 1. For phospho-ERK and phospho-Akt, values were normalised first to β-actin/ HSP90 and then to total ERK and total Akt respectively.

Intracellular staining for flow cytometry

Trypsinised cells were pelleted at 400*g* for 5 min, washed once with PBS, re-suspended in 4% paraformaldehyde and incubated for 20 min at room temperature. Next, cells were washed with PBS and resuspended in permeabilisation buffer (0.1% Triton-X100 in PBS) for 5 min. After another wash with PBS, cells were incubated in 0.5 ml blocking buffer (2% BSA in PBS). Primary antibody (α-SMA; 1A4; Sigma) was added to the blocking buffer at a dilution of 1:500. After a further incubation of 1h at room temperature, cells were washed twice with PBS, resuspended in blocking buffer containing 1:500 diluted fluorescent secondary antibody (Alexa 488-conjugated anti-mouse; cat no. A11011; Life

Technologies) and incubated for 1h in the dark. Following another two washes with PBS, cells were analysed using FACS-Calibur (BD Biosciences).

TEM

Briefly, 10 µl EVs solubilised in ultrapure water were dropped on to Parafilm (cat no. PM998; Bemis). A carbon coated formvar copper grid (cat no. TG50Cu; EM Resolutions) was placed on the droplet to immerse its coated side, and incubated for 30s at room temperature. Excess sample was blotted away using absorbent paper. The grid was then incubated with 10 µl negative stain (5% ammonium molybdate/ 1% trehalose) for 10s. Excess negative stain was removed by blotting. The grid was visualised at increasing magnification up to 135 000x using the Tecnai 12 microscope (FEI).

Nanoparticle Tracking Analysis (NTA)

The size distribution of EVs was measured by NTA (NS300; NanoSight), equipped with an EMCCD camera and a 405nm diode laser. Silica beads (100 nm diameter; cat no. 140120-10; Microspheres-Nanospheres) were used to calibrate the instrument. EV samples were diluted 1:5000 in PBS to optimise the particle number in the field of view. For each sample, five videos, each of 90s duration, were captured. Analysis was performed using the instrument software (NTA 2.3.0.15).

RNA extraction

Cellular RNA was isolated using the miRNeasy mini kit (cat no. 217004, Qiagen) and EV RNA using the miRNeasy micro kit (cat no. 217084; Qiagen), as per the manufacturer instructions. RNA quality and concentration was determined using the NanoDrop 8000 spectrophotometer (ThermoFisher).

MiRNA array

The Cancer MicroRNA qPCR Array with QuantiMir™ (cat no. RA610A-1; SBI System Biosciences) was used to profile CRC cells and EVs for 95 cancer-related miRNAs, as per manufacturer instructions. Samples were processed in triplicate. A global mean normalisation method was used, as previously described (D'Haene et al. 2012). Combined mean $2^{(-\Delta\Delta CT)}$ values for epithelial cells or EVs (DLD-1, HCT116 and SW620) were compared with mesenchymal (SW480) cells or EVs for each miRNA, to

generate fold changes. MiRNAs for which $2^{(-\Delta\Delta CT)}$ values were less than 0.1 in all samples were excluded. EV miRNA profiling data was deposited in Exocarta (Mathivanan et al. 2012).

RT-qPCR for miR-200

Total RNA was converted into cDNA using the Taqman Advanced miRNA cDNA Synthesis Kit (cat no. A28007; ThermoFisher). Input material was 4ng of total RNA (2 µl of 2 ng/µl). Expression levels were normalised to miR-423-5p (endogenous reference gene; based on literature (Link, Krohn, and Schumann 2019; Bignotti et al. 2016) and our own observations (**Figure 3**)), calculated from the triplicate of CT values, using the ΔΔCT method, and expressed relative to one of the samples that was assigned the value 1. Mean relative levels were calculated for each sample. Assay reference numbers were as follows: miR-200a-3p (478490_mir), miR-200b-3p (477963_mir), miR-200c-3p (478351_mir) and miR-141-3p (478501_mir). Mature miRNA sequences are shown in Table S1. For PCR reaction, the miR-Amp product was diluted 1:10 with RNase-free water, and 5 µl added to one well of a 96-well plate. This was combined with 10µL TaqMan® Fast Advanced Master Mix (2X), 1 µl TaqMan® Advanced miRNA Assay (20X) and 4 µl RNase-free water (total reaction volume 20 µl). Reactions were set up in triplicate with the following cycling conditions: 95°C for 20s, followed by 40 cycles of 95°C for 3s/ 60°C for 30s, using the Applied Biosystems 7500 qPCR instrument.

Table S1. Taqman Advanced miR-200 qPCR assays (Applied Biosystems/ ThermoFisher)

MiRNA	Assay ID	Cat no.	Mature sequence
miR-200a-3p	478490_mir	A25576	UAACACUGUCUGGUAACGAUGU
miR-200b-3p	477963_mir	A25576	UAAUACUGCCUGGUAAUGAUGA
miR-200c-3p	478351_mir	A25576	UAAUACUGCCGGGUAAUGAUGGA
miR-141-3p	478501_mir	A25576	UAACACUGUCUGGUAAAGAUGG
miR-423-5p	478090_mir	A25576	UGAGGGGCAGAGAGCGAGACUUU

MiR-200 inhibitor transfection of CRC cells

DLD-1 cells (2 x 10^6) were seeded into 10cm dishes (day 0). The following day (day 1), cells were transfected with miR-200 inhibitor (antagomiR) or control RNA (see below) and passaged after 6h to

three 15 cm dishes in EV-free media. On day 5, DLD-1-control- and antagomiR-transfected EVs were isolated and used to condition fibroblasts.

MiRNA inhibitors (500 pmol, combined miR-200a/b/c and -141) or scrambled sequence control were transfected using Lipofectamine 2000. MISSION miR-200a-3p inhibitor (cat no. HSTUD0350; Sigma), miR-200b-3p inhibitor (cat no. HSTUD0352; Sigma), miR-200c-3p inhibitor (cat no. HSTUD0354; Sigma) and miR-141-3p inhibitor (cat no. HSTUD0216; Sigma) and MISSION siRNA universal negative control #1 (cat no. SIC001; Sigma) were used. Transfection efficiency was determined 48h after transfection by RT-qPCR.

MiRNA, antagomiR and siRNA transfection of fibroblasts

MRC5 or NCFs (4 x 10^5 cells) were seeded into 6-well plates (day 0). The following day (day 1), cells were transfected with miR-200 mimics, *ZEB1* siRNA or scrambled sequence control RNA using Lipofectamine 2000. Six hours after transfection, cells were split 1:2. On day 3, medium was switched to low serum medium (0.1% FBS), and on day 4, TGF-β (2 ng/ml) was added to half the wells (the remainder were controls). On day 6, cells were collected for western blotting.

100 pmol miRNA mimic, antagomiR or siRNA were transfected using Lipofectamine 2000 (as described above). MISSION miR-200b-3p (cat no. HMI0352; Sigma) and miR-200c-3p (cat no. HMI0354; Sigma) mimics, MISSION miR-200b-3p inhibitor (cat no. HSTUD0352; Sigma), Hs_ZEB1_6 FlexiTube siRNA (cat no. SI04951072; Qiagen) and MISSION siRNA universal negative control #1 (cat no. SIC001; Sigma) were used. Transfection efficiency was determined 48h after transfection, by RT-qPCR for miR-200b and -200c, or western blotting for Zeb1.

Immunocytochemistry

5 x 10^3 fibroblasts were plated for immunocytochemistry on permanox chamber slides (cat no. 177402; ThermoFisher), fixed in 4% PFA for 20 min and then permeabilised with 0.5% TritonX-100 (in PBS) for 10 min. After blocking for 2h with 2% BSA/ 0.1% TritonX (in PBS), primary antibody incubation was performed with anti-SMA (A2547, 1:750, Sigma) for 60 min followed by secondary antibody incubation with AlexaFluor 568 antibody (cat no. A21144; ThermoFisher) for 60 min. DAPI was used

as nuclear counterstain and coverslips were mounted using Fluorescent Mounting Medium (cat no. S3023; Dako). Images were taken using an Olympus IX81 fluorescence microscope and Xcellence program. To avoid x-y bias, the centre of the slide was imaged and all cells in the image were included in the analysis. Fluorescence intensity for each cell was determined using ImageJ and data points from three independent experiments were presented.

Luciferase reporter assays

2 x 10^4 MRC5 fibroblasts, were seeded into each well of a 96-well plate. The following day, each well was transfected with 15 ng Firefly, 15 ng mCherry, 200 ng luciferase reporter construct (ZEB1 3'UTR, 200b mutant 3'UTR or control 3'UTR) and 2 pmol miRNA mimic or scrambled sequence control, using 0.5 µl Lipofectamine 2000 (cat no. 11668027; ThermoFisher) in 100 µl OptiMEM (cat no. 31985070; ThermoFisher). After 24h, transfection efficiency was assessed by detection of mCherry signal by fluorescence microscopy. The medium was then changed to regular DMEM. Cells were then left for a further 24h before detection of luciferase activity.

Firefly and Renilla luciferase activity were measured using the Dual-Glo® Luciferase Assay System (cat no. E2940; Promega). Cells were first equilibrated room temperature. 100 µl Luciferase Assay Reagent was added to each well and incubated for 10 min on a rocker at room temperature. Firefly luciferase activity was then measured using a luminometric plate reader (Varioskan LUX Multimode Microplate Reader; ThermoFisher). 100 µl Stop & Glo Reagent was then added to each well and incubated for 10 min on a rocker at room temperature. Renilla luciferase activity was then measured on the same plate reader. For each well, Firefly activity was normalised by Renilla activity.

In vivo study

PKH26 labelling of fibroblasts: PKH26 labelling was conducted as per manufacturer recommendations using the PKH26 fluorescent cell linker kit (cat no. PKH26GL; Sigma). Final concentrations after mixing the indicated volumes were 1 × 10^7 cells/ml and 2 × 10^{-6} M PKH26. After intermittent mixing for 5 min, 10 ml of complete DMEM was used to stop the reaction. Labelled cells were centrifuged at

400*g* for 10 min at 20-25°C and the supernatant discarded. Cells were washed with complete DMEM twice before being resuspended in the appropriate volume for use.

Immunohistochemical staining of mouse tumours: Three tumours from each of the groups (mesenchymal and epithelial), were fixed overnight at room temperature in 10% neutral buffered formalin, then sectioned and stained with: (i) haematoxylin and eosin; (ii) Zeb1 (sc25388; Santa Cruz Biotechnology; 1:500); (iii) α-SMA (1A4; Dako; 1:200). Quantification of immunohistochemical staining was done by a histopathologist. Images were divided into 100 μm^2 squares (gridded screen) and the central nine squares of each image were analysed for positively and negatively stained CRC cells and fibroblasts. Optical Density (OD) calculator was used as a digital pathology platform. Signal localisation (nucleus-cytoplasm) was considered when identifying positive staining.

Dissociation of tumours into single cells: The remaining tumours (nine from each group) were pooled together and dissociated into single cells for flow-sorting. Tumours were collected in ice-cold DMEM/F12 medium (cat no. 21331020; Gibco/ ThermoFisher) and then sterilised by immersion in 70% ethanol for 5s, followed by washing in two changes of regular DMEM. Tumours were then cut into fragments of 1 mm^3 and incubated at 37ºC for 2h with the digestion solution (0.125% collagenase (cat no. 10103586001; Roche); 0.0125% hyaluronidase (cat no. H3506; Sigma); 1 mM L-glutamine; 5% FBS; in 10ml DMEM/F12). The tissue was agitated every 30 min by vortexing for 1s. After 2h, the tissue was vortexed for 5s and then centrifuged at 350*g* for 5 min. The pellet was resuspended in 30 ml of HBSS (containing 2% FBS) and centrifuged at 350*g* for 5 min. This was repeated with HBSS without serum. The pellet was then resuspended in 5ml pre-warmed 0.25% Trypsin-EDTA (cat no. T4049; Sigma) and continuously mixed for 2 min by pipetting, to mechanically dissociate the organoid-like structures. 30ml of HBSS (+ 2% FBS) was added and the cell suspension centrifuged at 350*g* for 5 min. 2 ml of 5 mg/ml pre-warmed Dispase II (cat no. 4942078001; Roche), containing 20 µl of 10 mg/ml DNase I (cat no. 10104159001; Roche) was then added to the cell pellet. The cells were then pipetted up and down for 2 min to dissociate the remaining clumps. 30 ml of cold HBSS (+2% FBS) was then added and the cells centrifuged at 350*g* for 5 min. The pellet was resuspended in HBSS (+2% FBS) and filtered through a 40 µm cell strainer into a new tube. This was then centrifuged again at 350*g* for 5 min

and the supernatant discarded. The final cell pellet was resuspended in regular DMEM and taken for flow-sorting.

Sorting of PKH26-positive and –negative cells: Cells were sorted on FACS Aria (BD Biosciences). Instrument parameters were set up using unlabelled MRC5 fibroblasts, after which samples of interest were sorted. First, debris and apoptotic cells were gated out. Next, additional gates were drawn in the PKH26 and forward scatter channels to select viable PKH-positive cells. Cells registered above the control gate were considered PKH-positive. Specific parameters were as follows: 488 nm (blue) laser; Neutral Density Filter 1.0; Longpass Mirror 556LP; Bandpass Filter 576/26. Cells were collected in 15 ml Falcon tubes containing DMEM/ 10% FBS, which had been previously coated with FBS at 4°C for 1h. Immediately after sorting, cells were centrifuged at 500*g* for 5 min and the pellet stored at -80°C, prior to quantification of miR-200, *ACTA2* and *FN1* levels.

RT-qPCR for ACTA2 and FN1: The miScript II RT kit (cat no. 218160; Qiagen) was used to generate cDNA for mRNA quantitation, as per manufacturer instructions. SYBR Green RT-qPCR assays were performed to quantitate *ACTA2* and *FN1* expression in PKH-positive cells. Reactions were set up in 96-well plates. Each well contained 12.5 µl of 2x QuantiTect SYBR Green PCR Master Mix (cat no. 204141: Qiagen), 2.5 µl 10x miScript Primer Assay, 7.5 µl of RNase-free water and 2 µl of cDNA template (total reaction volume 25 µl. Reactions were set up in triplicate with the following cycling conditions: 95°C for 15 min, followed by 40 cycles of 94°C for 15s/ 55°C for 30s/ 7°C for 34s, using the Applied Biosystems 7500 qPCR instrument. Expression levels were normalised to *GAPDH* (endogenous reference gene) calculated from the triplicate of CT values using the ΔΔCT method, and expressed relative to mesenchymal (SW480 control) tumours, which were assigned the value 1. Mean relative levels were calculated for each sample. The miScript Primer Assays were: Hs_ACTA2_1_SG QuantiTect Primer Assay (product no. QT00088102; Qiagen), Hs_FN1_1_SG QuantiTect Primer Assay (product no. QT00038024; Qiagen) and Hs_GAPDH_1_SG QuantiTect Primer (product no. QT00079247; Qiagen).

Supplementary References

Bhome, R., R. W. Goh, M. D. Bullock, N. Pillar, S. M. Thirdborough, M. Mellone, R. Mirnezami, D. Galea, K. Veselkov, Q. Gu, T. J. Underwood, J. N. Primrose, O. De Wever, N. Shomron, A. E. Sayan, and A. H. Mirnezami. (2017). Exosomal microRNAs derived from colorectal cancer-associated fibroblasts: role in driving cancer progression, *Aging (Albany NY)*, *9*(12): 2666-94.

Bignotti, E., S. Calza, R. A. Tassi, L. Zanotti, E. Bandiera, E. Sartori, F. E. Odicino, A. Ravaggi, P. Todeschini, and C. Romani. (2016). Identification of stably expressed reference small non-coding RNAs for microRNA quantification in high-grade serous ovarian carcinoma tissues, *J Cell Mol Med*, *20*(12): 2341-48.

D'Haene, B., P. Mestdagh, J. Hellemans, and J. Vandesompele. (2012). miRNA expression profiling: from reference genes to global mean normalization, *Methods Mol Biol*, *822*: 261-72.

Li, M. Z., and S. J. Elledge. (2012). 'SLIC: a method for sequence- and ligation-independent cloning', *Methods Mol Biol*, *852*: 51-9.

Link, F., K. Krohn, and J. Schumann. (2019). Identification of stably expressed housekeeping miRNAs in endothelial cells and macrophages in an inflammatory setting, *Sci Rep*, *9*(1): 12786.

Mathivanan, S., C. J. Fahner, G. E. Reid, and R. J. Simpson. (2012). ExoCarta 2012: database of exosomal proteins, RNA and lipids, *Nucleic Acids Res*, *40*(Database issue): D1241-4.
